# On the Role of Polymer Viscoelasticity in Enhanced Oil Recovery: Extensive Laboratory Data and Review

**DOI:** 10.3390/polym12102276

**Published:** 2020-10-03

**Authors:** Alexander Rock, Rafael E. Hincapie, Muhammad Tahir, Nils Langanke, Leonhard Ganzer

**Affiliations:** Institute of Subsurface Energy Systems, Clausthal University of Technology, 38678 Clausthal-Zellerfeld, Germany; alexander_rock@gmx.de (A.R.); muhammad.tahir@tu-clausthal.de (M.T.); nils.langanke@tu-clausthal.de (N.L.); leonhard.ganzer@tu-clausthal.de (L.G.)

**Keywords:** hydrolyzed polyacrylamides, viscoelasticity, oil recovery

## Abstract

Polymer flooding most commonly uses partially hydrolyzed polyacrylamides (HPAM) injected to increase the declining oil production from mature fields. Apart from the improved mobility ratio, also the viscoelasticity-associated flow effects yield additional oil recovery. Viscoelasticity is defined as the ability of particular polymer solutions to behave as a solid and liquid simultaneously if certain flow conditions, e.g., shear rates, are present. The viscoelasticity related flow phenomena as well as their recovery mechanisms are not fully understood and, hence, require additional and more advanced research. Whereas literature reasonably agreed on the presence of these viscoelastic flow effects in porous media, there is a significant lack and discord regarding the viscoelasticity effects in oil recovery. This work combines the information encountered in the literature, private reports and field applications. Self-gathered laboratory data is used in this work to support or refuse observations. An extensive review is generated by combining experimental observations and field applications with critical insights of the authors. The focus of the work is to understand and clarify the claims associated with polymer viscoelasticity in oil recovery by improvement of sweep efficiency, oil ganglia mobilization by flow instabilities, among others.

## 1. Introduction

### 1.1. Overview of Polymer-Enhanced Oil Recovery

Although oil production is currently assumed to be higher than the actual demand, the future of oil and gas production will have to tackle severe challenges: fewer new fields are found and major producing fields show declining production. Most of the latter fields traversed all three production phases of an oil reservoir. A primary recovery phase using the natural energy present in the reservoir can reach recovery factors (RF) of up to 30% [1]. A secondary production phase using artificial methods such as pressure support by water flooding or the application of pumps can yield RFs of approximately 50–60% [1,2]. The last phase of recovery—the tertiary recovery phase enhanced oil recovery (EOR) methods—is applied in order to achieve RFs of up to 80% [3,4].

Currently, the modern oil and gas industry possess several EOR options that could be applied, such as thermal methods (often for heavy oil recovery) by introducing additional heat into the reservoir [5], miscible processes with injection of CO_2_ for example [6] and chemical applications such as polymer flooding, surfactant flooding and combined processes, e.g., alkaline-surfactant-polymer flooding [7]. 

In a more specific sense, polymer flooding has proven to be an excellent process economically speaking as a well-recognized and widely used chemical EOR method. This is mainly due to the large number of advanced and extensive experimental investigations (e.g., [2,8,9,10,11,12,13,14,15] as well as due its success in field applications such as the Daqing oil field in China [16]. The remarkable potential of polymer EOR is additionally underlined by Sheng et al. [17] who analyzed 733 polymer flooding projects around the world. The compilation shows that the oil recovery utilizing polymer injection results in a 6.7% higher recovery than using conventional water flooding. 

Several mechanisms are being used to explain the oil recovery due to polymer process application in reservoirs. Although, there appears to be a sort of consensus, researchers keep on searching for clear explanations and answers. The mechanisms behind the observed recovery factors that can be found in the literature are manifold but can be grouped as follow in Table 1.

Although polymer flooding offers an extensive variety of recovery mechanisms and shows remarkable success in field applications, it comes with some considerable technical and economic challenges. First of all, polymer flooding projects require significant planning and testing for successful field implementation, e.g., laboratory investigations, pilot tests and reservoir simulations [10,16,32,33,34]. 

Additionally, polymer solutions require an optimized fluid chemistry in order to have the desired viscosity in the reservoir or rather a mobility ratio of the whole fluid system. Hereby, polymer EOR applications have to consider reservoir depth, reservoir temperature, reservoir thickness, porosity, permeability, oil gravity, oil viscosity and water chemistry (especially salinity and hardness) [35]. All these factors can decrease the quality of the injected polymer solutions which potentially results in an unfavorable polymer solution viscosity. In turn, this can result in economical as well as technical unfeasibility. These geological and fluid characteristics do not only influence the viscosity of the injected polymer solution, but also the viscoelasticity which is believed to contribute to the oil recovery as well. 

In situ rheology is the name given to the related aspects of rheology of polymeric solutions as they flow through porous media. EOR polymers, depending on the rate or time of deformation, can either store energy (an elastic response), dissipate energy (viscous response), or both. This is often termed as the shear-thinning and shear thickening behaviour in rheological terms. As previously mentioned, EOR viscoelastic polymers can also exhibit notorious resistance to stretching (high extensional viscosity) compared to the behaviour obtained when polymers are only under shear. EOR polymers, which have been utilised lately for flooding applications and which tend to be high molecular weight polymers (such as partially hydrolysed polyacrylamides (HPAM) along with biopolymers). Note that according to literature and the later shown data in this work, the viscoelastic property is most likely to be depicted with high molecular weight polymers and seemly high concentrations. Due to this property, as well as solution concentrations, these polymer solutions may show a combination of viscous and elastic properties, known as viscoelasticity. This becomes of high importance since the fluid is forced to change its own speed to be able to maintain a fixed volumetric rate while crossing over the pores. As a result, extensional forces are generated in the flow path simultaneously with the fluid being under shear forces close to the wall areas. All these properties are best described and defined by different devices or rheometer that relate the connection between the stress (forces) and strain rate (deformation) tensors under different flow conditions. The constitutive equations that are often applied in the field of continuum mechanics are also useful.

We present data that cover the areas of shear thinning, shear thickening and elastic turbulence, all associated to the interpretations of the possible existence of viscoelastic properties/behaviour. An additional element is the elastic turbulence, which differs from shear thickening. Reported results by Hincapie et al. [29], Rock et al. [30], Groisman and Steinberg [31] and other authors, point out that at low Reynolds numbers, elastic turbulence occurs. Polymers with high elasticity change the stability of the laminar flow causing elastic instabilities. This means the elastic turbulence is likely to occur at the pore level and represents a more recent interpretation on the effects in porous media. 

Overall, this shows the necessity of polymer solution optimization and especially, the requirement for a viscoelasticity-focused optimization approach. In order to account for the latter, it is imperative to understand the EOR polymers’ viscoelastic behavior in all its different shades.

### 1.2. Scope of the Study

The present work aims to provide an extensive review of the importance of polymers’ viscoelasticity (if depicted) and their associated flow behavior in EOR applications. This helps to optimize the aqueous polymer solutions injected in oil reservoirs, develop and test new viscoelastic polymers, understand their degradation in the reservoir, their recovery mechanisms and finally, be able to assess the viscoelasticity’s importance in polymer flooding. This is achieved by reviewing and discussing the following aspects:

- Effect of polymer viscoelasticity, different polymer chemistries, polymer molecular weight distributions and concentrations on the oil recovery;
- Effect of reservoir properties on polymer viscoelasticity and its related recovery efficiency;
- Effect of thermal, chemical, mechanical and chemical degradation on the polymer’s viscoelastic flow behavior;
- Contribution and importance of the viscoelastic flow characteristics on EOR.

In a general sense, this work has the scope to manifest the importance and potential of viscoelasticity in polymer EOR applications and, hence, underline the need for further comprehensive investigations.

### 1.3. Paper Organization

This work is arranged in seven sections. The first section provides a brief and general introduction in polymer flooding and the role of fluid viscoelasticity in it. The second section gives an overview about the different polymer types used in EOR as well as a discussion on their properties. Subsequently, the third section represents an extensive review on the role of viscoelasticity in EOR applications. Within this section, a definition of viscoelasticity and an overview of experimental approaches for its investigation are given. Furthermore, the third section provides a discussion on the physics and mechanics behind viscoelasticity and specifically on the viscoelastic flow phenomena observed in porous media. Moreover, the chemistry of viscoelastic polymers and the impacts on their flow behavior is reviewed. The following fourth section aims to critically discuss the viscoelastic recovery mechanisms and their importance on EOR. In the fifth section, the importance of viscoelasticity in polymer EOR applications, its potential, synergies and impacts are summarized. Following this, a summary and detailed conclusion of the study are given. 

## 2. Polymers for Enhanced Oil Recovery (EOR): Types and Chemistry

Although a wide range of polymers is available for EOR applications, there are basically two main types of polymer: first, synthetic polymers (e.g., polyacrylamides) including a large variety of modifications in its chemistry; and second, biopolymers which are mainly polysaccharides that are obtained by biological processes at an industrial scale.

### 2.1. Synthetic Polymers

**Figure 1:**
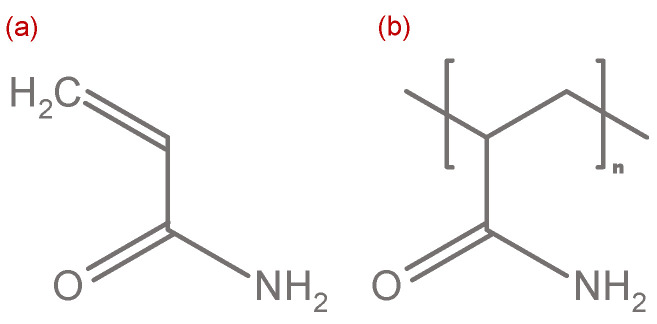
(**a**) Acrylamide monomer used for polymerization of polyacrylamides (PAMs) (modified after Lentz et al. [39]; (**b**) PAM chain after polymerization (modified after Hotta et al. [40].

Synthetic polymers are extensively used in EOR applications worldwide due to good understanding of the polymer system (e.g., inter- and intramolecular interactions and recovery mechanisms), their availability and relatively low costs [36]. One of the simplest and most widely used synthetic polymers used in the oil and gas industry are polyacrylamides (PAMs) [18,37]. Polyacrylamides consist of multiple acrylamide monomers. An acrylamide molecule is shown by Figure 1a. It can be seen that an acrylamide monomer consists of a hydrocarbon chain with a double-bound oxygen atom and an amino group at one end of it. In order to generate viscosifying polyacrylamides these monomers needs to be polymerized. A common practice for polymerization of acrylamide monomers is the use of ammonium persulfate as initiator and a catalyst, namely tetramethylethylenediamine [38]. The resulting PAM is shown in Figure 1.

A linear PAM as shown in Figure 1b has no significant gel strength which means that their viscosifying characteristics is degraded when experiencing mechanical stress such as shearing in the porous reservoir rock [41]. Therefore, linear PAMs are additionally cross-linked in order to improve their shear resistance which has a positive effect on the injectivity. For this purpose, methylenebisacrylamide is used in the polymerization process [38]. 

**Figure 2:**
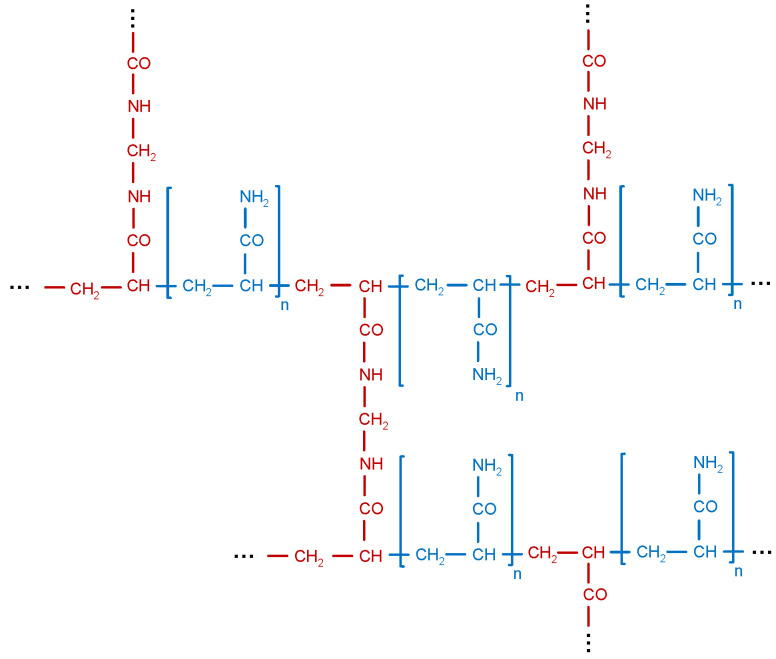
Cross-linked PAM (red = methylenebisacrylamide, blue = PAM) (modified after National Diagnostics [45]).

The exemplary structure of a cross-linked PAM used in EOR applications is shown by Figure 2. It can be seen that the red methylenebisacrylamide connects and cross-links the linear PAMs, thereby creating a three-dimensional complex structure. This complex structure enables the PAM to withstand higher mechanical stresses which is important during the flow through porous oil reservoirs, especially in cases where permeability is rather low. Other cross-linking agents commonly used are chromium (inorganic), phenol, formaldehyde, polyethylenimine and chitosan (all organic) [42]. Generally, the decision as to which HPAM chemistry should be chosen is strongly dependent on different reservoir and fluid effects. Divers et al. [43] and Gaillard et al. [44] give detailed insights for definition and decision of the proper polymer type in different applications. Although PAMs are relatively good viscosifying agents, further modifications are made in order to increase its applicability in EOR. A very important characteristic of synthetic PAMs is their viscoelasticity which is explained in detail in Section 3. In order to increase the viscoelasticity of a PAM, the amide groups of the PAMs can be hydrolyzed to a certain degree, forming a partially hydrolyzed polyacrylamide (HPAM). 

**Figure 3:**
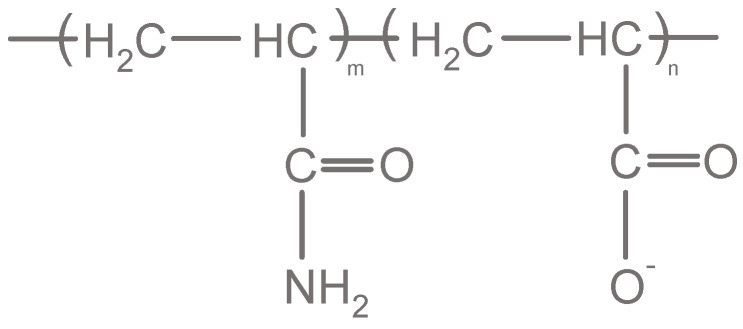
Molecule of a partially hydrolyzed polyacrylamide (modified after Zhu et al. [46]).

An exemplary HPAM molecule structure is shown in Figure 3. HPAMs are widely used in oil-recovery applications due to their excellent solubility characteristics in water and their strong thickening ability [46]. Nevertheless, the hydrolysis of PAMs transforms the polymer into a polyelectrolyte with negative charges which in turn react with cations of the reservoir brine [42]. This leads to a high sensitivity of HPAMs against solution water salinity and is one of the major issues regarding the HPAM stability. The degree of hydrolysis does not only influence the viscoelasticity and the overall stability of polymer solutions, but also the viscosity in general. Kulicke and Hörl [47] have shown that the viscosity increases between a hydrolysis of 30–70% and decreases accordingly for a hydrolysis below 30% and above 70%. Spildo and Sae [48] explain the hydrolysis-related viscosity increase with an increase in repulsive forces yielding to polymer conformation which takes up a higher volume and thereby increases the viscosity of the solution.

Another chemical factor influencing not only viscoelasticity but also the viscosity of aqueous polymer solutions is the charge density. Spildo and Sae [48] have shown that for a HPAM with equal molecular weight and hydrolysis degree but different charge densities, the viscosity as well as the viscoelasticity vary. Hereby, the polymer solution with a broad charge distribution has shown a lower viscosity/viscoelasticity whereas the one with a narrower distribution had a higher viscosity/viscoelasticity in comparison. 

Apart from charge density and hydrolysis, also the molecular weight has an influence on the viscosity or rather viscoelasticity. This can be explained by the previous argument of Spildo and Sae [48] once again. In order to create a polymer molecule with a high molecular weight it is necessary to add up single PAM molecules to a long chain and if the length—thereby the number of PAMs—increases the molecular weight increases due to an increasing volume occupation. Furthermore, a longer polymer chain has more hydrolyzed backbones which increase the fluid interactions. 

Overall, HPAM solutions have promising viscosifying and viscoelastic characteristics, but are also very sensitive to salt, mechanical stress and temperature. Therefore, hydrophobic association polymers (HAPAMs) were invented to overcome the disadvantages of HPAM solutions. HAPAMs are PAMs to which hydrophobic side-chains are connected. This not only decreased HPAMs/PAMs sensitivity against salinity and mechanical degradation, but also improved the polymer solution properties such as an enhanced viscosity, fluid–fluid interactions and also the ability to form emulsions. Although HAPAMs show encouraging results in laboratory measurements, it is important to mention that these polymers have not been studied in field applications yet and, therefore, there is a lack of understanding about them [49].

Another modification of PAMs is KYPAM which is an aromatic hydrocarbon monomer with an ethylene group. Thereby, KYPAM has a higher resistance against salt and because of the repulsion between its hydrophobic groups -resulting in stretched polymer chains- a higher viscosity compared to HPAMs [17].

In addition to a PAM and its different modifications, another synthetic polymer is 2-acrylamide-2-methyl propanesulphonate (AMPS) which is made up of anionic sulphonate protecting the acrylamide groups of its molecule and yielding to a good stability in high salinity brine. The acrylamide groups of AMPS lead to a good thermal stability as well as to a remarkable resistance against acidic and alkaline environments and hydrolysis [17].

### 2.2. Biopolymers

Another type of polymer which is more often used in EOR application currently are biopolymers. These polymers are used because they are not officially listed as chemicals and, therefore, can be applied more easily, even in countries with very strict health, safety and environment regulations. Since this study aims to provide an extensive review on the viscoelasticity of polymers in EOR applications, biopolymers are not explained in detail here due to their missing viscoelastic properties. Nevertheless, a small paragraph is dedicated to this kind of polymer in order to complete the list of different polymer types. 

A major difference between synthetic and biopolymers is their origin. Whereas HPAMs are synthesized from monomers, biopolymers are typically obtained by fermentation in which bacteria produce the polymer as a result of their metabolic processes. The most widely used bacteria is *Xanthomas campestris* from which the biopolymer xanthan is derived. Another recently discussed biopolymer is schizophyllan which is also a polysaccharide [50]. The molecular weight of biopolymers is low in comparison to synthetic polymers which results in a relatively rigid molecule structure. As a result of this, biopolymers are excellent viscosifying agents which can be used in high-salinity water. Additionally, biopolymers show remarkable shear resistance due to their rigid structure. A disadvantage of these polymers is the high sensitivity to thermal degradation as well as the lack of polymer retention on the rock surface which means that there are no resistance effects after polymer flooding. Furthermore, biopolymers, due to their low molecular weight and missing hydrolyzed backbones, show no viscoelastic properties which can be understood as a significant disadvantage in EOR applications as the results from Hincapie et al. [29] and Needham and Doe [51] indicate.

## 3. Viscoelasticity in Enhanced Oil Recovery

This section provides an extensive review on the viscoelastic flow phenomena encountered during the flow in porous media, e.g., in EOR applications. It gives a definition of viscoelasticity itself, the different experimental studies performed in literature and the mechanics during the viscoelastic fluid flow. Furthermore, the various impacts on the polymers’ viscoelasticity are discussed in detail in order to understand and outline the necessity of polymer solution optimization in enhanced oil recovery. 

The key aspects in optimising polymer rheology for EOR applications need to be examined in order to improve process performance. Until recently, research investigating the complementarity of different rheological techniques to fully characterise polymers used for EOR has been surprisingly neglected. As previously described by Hincapie [8] and Hincapie and Ganzer [52], non-linear viscoelastic experiments measured on a rotational rheometer can be used to provide an explanation for viscoelastic polymer behaviour related to porous media and to determine the influences of the environmental conditions. Different evaluations allow the possible determination of polymer viscoelastic properties from a rheological point of view. The measurements described include steady shear viscosity, small amplitude oscillatory shear (SAOS, stress relaxation), N_1_ (streamline tension) and extensional thickening (strain hardening). This is to demonstrate the complementarity of different rheological techniques to fully characterise polymers used for EOR. Moreover, it depicts how non-linear viscoelastic measurements performed on a rotational rheometer can be a useful tool to relate the behaviour of viscoelastic polymers to that in porous media. 

Evaluations are often performed taking into account the different effect of degradation, named thermal and mechanical, often described in the literature. 

### 3.1. Viscoelasticity as Per Definition

**Figure 4:**
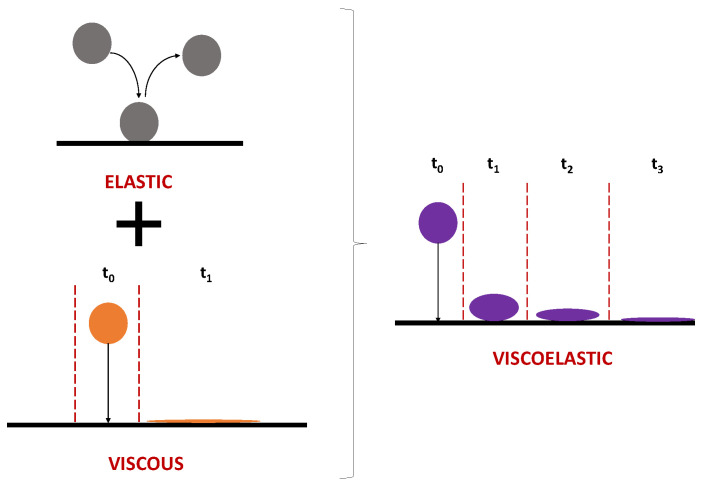
Illustration of elastic, viscous and viscoelastic behavior.

In order to understand the concept of viscoelasticity it helps to first define the standard equation for an incompressible, isotropic Newtonian fluid in which the shear viscosity is related to shear stress and rate by;

(1) $$\eta =\frac{\tau }{\gamma }$$

where *η* is the shear viscosity in Pa.s, *τ* is the shear stress in Pa and *γ* is the shear rate in s^−1^ [53]. For a Newtonian fluid this relation is linear proportional. In comparison to a non-viscoelastic, Newtonian fluid, the correlation shown by Equation (1) is non-linear for a viscoelastic fluid, e.g., an aqueous HPAM solution. The non-linearity of shear viscosity, shear stress and rate are the result of the viscoelastic fluids’ ability to store (elastic behavior) and dissipate energy (viscous behavior) when the fluid molecule undergoes deformation, such as during the flow through narrow pore channels in reservoirs [30]. Furthermore, if a completely viscous fluid is deformed stress immediately reduces to zero once the strain is constant [54]. For a completely elastic solid no relaxation would be observed. Figure 4 illustrates the concept of the instantaneous relaxation of a Newtonian fluid and the time-dependent relaxation of a fluid which has elastic and viscous characteristics.

This relaxation behavior can also be translated into a relaxation modulus:

(2) $$G\left(t\right)=\frac{\tau \left(t\right)}{\gamma }$$

where *G* is the relaxation modulus in Pa [53]. Some researchers, e.g., Hincapie and Ganzer [52], Hincapie [8], Macosko [54] and Mezger [53] additionally distinguish between an elastic modulus G’ (energy storage) and viscous modulus G’’ (energy loss) exists. Based on these two moduli the relaxation time λ can be determined which is accepted to be a characteristic measure to quantify viscoelasticity [55,56,57]. The correlation between stress relaxation and strain given by Equation (2) is linear for γ < 0.5. This is also known as the plateau modulus Ge or linear-viscoelastic behavior. For γ > 1, the correlation is not linear anymore and therefore, in the definition of viscoelasticity it is necessary to distinguish between linear and non-linear viscoelasticity [54].

#### 3.1.1. Linear Viscoelasticity

Linear viscoelasticity can be mathematically described by the model proposed by Macosko [54] which is:

(3) $$\tau =\int_{-\infty }^{t}\overset{N}{\underset{k=1}{\sum }}G_{k\;}e^{-\frac{t-t^{\prime }}{\lambda_{k}}}\;\dot{\gamma \left(t^{\prime }\right)dt^{\prime }}$$

where *t* is the time in s and *t’* the past time running from the infinite past −∞ in s. A problem of the linear viscoelasticity model is the simplified assumption of an ideal shear situation, which means that the actual shear tensor has only two components. Therefore, the model given by Equation (3) does not represent a three-dimensional viscoelasticity and because of that it is not capable of predicting the stresses normal to the two-dimensional shear plane. This shows that for very low shear rates the shear viscosity does not depend on the shear rate. However, this model is only applicable at for γ < 0.5. 

#### 3.1.2. Non-Linear Viscoelasticity

As discussed above, the model of linear viscoelasticity does not account for normal stresses and, hence, another model has to be introduced describing the so called non-linear viscoelasticity. This model accounts for γ > 1 with:

(4) $$G\left(\gamma ,t\right)=\frac{\tau \left(t,\;\gamma \right)}{\gamma }$$

Thereby, the normal stresses and torsion which can occur during shearing or rather flow of a fluid. Stress response calculations for non-linear viscoelastic fluids can be made by using differential equations such as the Maxwell-type (Equation (4)), Kaye–Bernstein–Kearsley–Zapas-type (K-BKZ) (Equation (5)) or the Lodge equation (Equation (6)). Due to their long and complex form their derivation is not part of this study but are explained in detail in Macosko [54]. However, this researcher proposed to use the neo-Hookean (Equation (7)) model for stress response modelling in the most cases:

(5) Maxwell−type:τ+λ ∇(τ)+f(τ,D)=2μD

(6) $$K-BKZ-type:\;\tau =\int_{-\infty }^{t}M\left[\phi_{1}\;B+\;\phi_{2}B^{-1}\right]dt^{\prime }$$

(7) $$Lodge:\;\tau =\int_{-\infty }^{t}M\;[\left(t-t^{\prime }\right)B\left(t,t^{\prime }\right)dt^{\prime }$$

(8) Neo−Hookean: τ=GB

where *M* is a memory function of the fluid depending on time $M\left(t\right)=-\frac{dG\left(t\right)}{dt})$, *ϕ*_1_ and *ϕ*_2_ are time derivates of the shear stress during relaxation at two consecutive time steps and *B* is a tensor describing the deformation of the fluid at any point [54].

#### 3.1.3. Normal Stress Difference, *N*_1_

Another important quantity in the description of fluid viscoelasticity is the normal stress difference *N*_1_ and *N*_2_ whereas only *N*_1_ is discussed in this paragraph due *N*_2_’s measurement complexity and empirical origin. *N*_1_ is the quantification of fluid elasticity during the flow. As mentioned before, viscoelastic fluids experience a three-dimensional deformation during the flow and, therefore, the deformation of the fluid is represented by a 3 × 3 tensor containing three normal stresses *σ*_xx_, *σ*_yy_, *σ*_zz_ and six shear stresses. It is important to note that in a viscous force-dominated flow only one shear stress is observed. Based on this 3 × 3 fluid deformation tensor it is possible to define the first normal stress difference as;

(9) $$N_{1}=\sigma_{\mathrm{xx}}-\sigma_{\mathrm{yy}}$$

whereas *N*_1_ is in Pa. Macosko [54] reports that the first normal stress difference is always positive for isotropic viscoelastic materials [58].

Hincapie [8] discussed the effect of *N*_1_ on the oil recovery in porous media. It is stated that *N*_1_ can be used as a parameter for the description of the elastic behavior of a polymer solution and that increasing oil recoveries can be observed for an increasing normal stress difference. Nevertheless, this observation has to be seen critically since it is not clear if the additional oil recovery is the result of an increased *N*_1_ or of other effects such as viscoelastic flow instabilities. It can be assumed that with increasing *N*_1_, the degree of elastic turbulence increases resulting in an incremental oil recovery.

#### 3.1.4. Weissenberg Number

In literature there are some dimensionless numbers used to describe the flow characteristics of viscoelastic properties. One of these numbers is the Weissenberg number *W*_i_ given by the following equation:

(10) $$W_{i}=\lambda \dot{\gamma }$$

The number describes the non-isotropic arrangement of the polymer under shear stress and by that, gives a quantification of the elasticity and non-linearity of the fluid’s mechanical properties. For *W*_i_ = 0, the fluid behaves only viscous whereas for *W*_i_ > 0, the fluid shows elastic characteristics as well [59]. The Weissenberg number increases towards the injection and production wells due to a high shear rate (up to 100 s^−1^) and decreases in the reservoir between the wells. It is important to mention that *W*_i_ is usually higher at the injection well rather than at the production well, because the polymer undergoes degradation in the reservoir and, hence, the polymer solution has a lower relaxation time than at the injection well.

#### 3.1.5. Deborah Number

Another important dimensionless number describing the fluid’s viscoelastic behavior is the Deborah number *D*e which is defined by:

(11) $$De=\lambda \;\frac{v_{s}}{L}$$

where *v*_s_ is the superficial flow velocity of the fluid in m/s and *L* the flow length along which the fluid flows in m. In addition to the previously defined Weissenberg number, the Deborah number allows for a quantification of the elastic strain [25,60]. In contradiction to the definition given by Howe et al. [25], Macosko [54] defines De as the ratio of relaxation time to the characteristic flow time t (*D*e = λ/t). Moreover, Clarke et al. [55] defines the deformation of the polymer solution as viscoelastic for *D*e > 1 and viscous for *D*e < 1. The Deborah number value can vary throughout the reservoir and is not as easy to predict as *W*_i_. First of all, the relaxation time of the polymer decreases from the injection well towards the production well due to polymer degradation. This would indicate that *D*e decreases along the flow path in the reservoir. Furthermore, with increasing flow path length the Deborah number decreases as well. But since *D*e is dependent on *v*_s_ as well fluctuations of *D*e have to be expected in the reservoir. This can be due to varying permeability and porosities along the flow path which results in locally different superficial velocities.

#### 3.1.6. M Parameter

Based on the previous definitions of *W*_i_ and *D*e, another dimensionless parameter can be defined. The so called parameter M additionally acknowledge the elastic stress and stream curvature caused by viscoelasticity. M is defined by [61]:

(12) $$M=\sqrt{Wi\;De}$$

### 3.2. Viscoelastic Flow Phenomena in Porous Media

The state-of-the-art literature recognizes and discusses three different flow phenomena occurring in porous media. The phenomena are believed to be related to the viscoelastic nature of the polymer solutions, and can be classified as [8,62]:

(a) Shear thinning;
(b) Shear thickening; and
(c) Elastic turbulence.

This section discusses these viscoelastic flow phenomena in detail.

#### 3.2.1. Shear-Thinning Behavior

**Figure 5:**
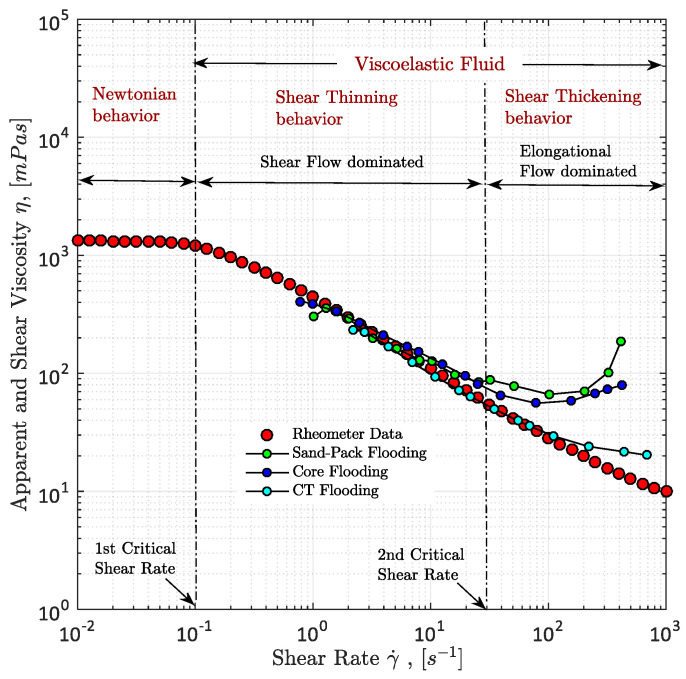
Viscoelastic flow behavior illustrated in a shear rate vs. apparent/shear viscosity plot.

As per rotational rheology definition, shear thinning is understood as a decreasing shear viscosity as a result of an increasing shear rate [54]. The behaviour can be seen from Figure 5. Before the viscoelastic behavior of a polymer solution takes off, a small plateau with a stable viscosity trend can be observed, which is referred to as a Newtonian fluid behavior. Usually Newtonian behavior occurs at very low shear rates. After the flow has reached the first critical shear rate, the shear-thinning behavior starts. In these regions of shear rates, the flow is dominated by shear. According to Macosko [54] shear thinning of viscoelastic polymers solutions can be explained by the molecular entanglement of the polymer. The viscosity of polymer solutions is also a result of entanglement. In order to generate entanglement of polymer molecules, the chains have to be close enough to each other and they have to be present in a defined volume for a finite time. With increasing shear rate, the polymer molecules’ motion relative to each other increases and as a result the density of entanglement is reduced, hence decreasing the polymer solution’s viscosity causing shear thinning behavior. It is important to mention that the critical shear rates shown in Figure 5 are only exemplary and can differ between different polymer types, solution properties as well as different porous media. The shear-thinning behavior was also experimentally investigated and extensively analyzed by Hincapie [8], Rock et al. [30], Tahir et al. [20,63] and Sheng [14] among many other researchers.

#### 3.2.2. Shear-Thickening Behavior

Apart from the shear-thinning behavior, usually a shear-thickening behavior can be observed for polymers injected in porous media, such as micromodels, sandstones or sand packs. Shear thickening can be generally defined as an increasing viscosity due to an increasing shear rate [64]. Shear-thickening behavior can be observed after the polymer flow has reached the second critical shear rate as shown in Figure 5. A remarkable observation that can be made from this plot is that shear thickening is only encountered in porous media during polymer flooding and not in rotational rheometer measurements [8,20,26,27,63,65,66,67,68]. It is believed that the missing shear thickening is the result of the missing flow motion, since the viscosity increase is commonly explained with a distinctive stretching of the polymer molecule [27,69]. Because of this stretching and the high shear rate, the polymer molecule has no sufficient time to re-coil itself. By that, the polymer flow is dominated by elongational forces and the viscosity increase is referred to as elongational viscosity or extensional viscosity [14,58,62,70,71,72]. 

Another explanation for shear thickening is given by Hincapie et al. [29]. The authors investigated the polymer flow in porous media quantitatively and qualitatively. By combining both input data, it was possible to relate the additional viscosity or rather the additional pressure drop along the porous media to viscoelastic flow instabilities. The streamline instabilities cause the polymer molecules to flow on a longer path through the porous media and thereby generating an additional differential pressure. So, as a consequence of the work of Hincapie et al. [29], shear thickening is actually not an increase in fluid viscosity, but only an additional pressure. Similar observations have been made by Clarke et al. [55], Tahir et al. [20,63] and Galindo-Rosales et al. [61].

#### 3.2.3. Elastic Turbulence

The most widely and recently discussed viscoelastic flow phenomena occurring in porous media is the so called elastic turbulence. Elastic turbulence can be defined as flow instabilities occurring due to the viscoelasticity of the injected polymer solutions. The first to describe the viscoelastic flow instabilities during the flow of aqueous polymer solutions were Groisman and Steinberg [31]. In their model, a step increase in the elastic stress is assumed to be the reason for the onset of elastic turbulence. The density of elastic stress of a viscoelastic flow can be estimated with *W*_i_ ν/2 (ν is the elastic stress) and was observed to be up to 65 times larger in the turbulent flow regime compared to the laminar flow. 

Furthermore, Groisman and Steinberg [31] describe the evolution of the viscoelastic flow instabilities. Firstly, and because of the extensive polymer molecule stretching, an unstable and irregular secondary flow is introduced. This secondary flow further influences the polymer molecules resulting in a further stretch which creates an even more distinctive flow instability until a complete irregular, dynamic flow is established in the porous media. Based on the work from Groisman and Steinberg [31] which mainly analyzed the elastic turbulence on a quantitative basis, other researchers performed experimental investigations with a more qualitative focus describing the instabilities’ characteristics.

Howe et al. [25], for example, investigated viscoelastic flow instabilities as well. They present streamline photographs made during their flow experiment in micromodels that show the elastic turbulence phenomenon. Furthermore, they show a correlation between the elastic turbulence and the onset of shear thickening of viscoelastic polymer solutions. Clarke et al. [55] also presented streamline images of elastic turbulent flow in micromodels. However, these images show a lack of quality not enabling a detailed description of the viscoelastic flow instabilities’ characteristics. 

Galindo-Rosales et al. [61] also performed experimental micromodel studies in order to investigate the elastic turbulent behavior of viscoelastic polymer solutions. For that, these researchers injected PAA solutions at various concentrations and flow rates into symmetric micromodels. Although they were able to observe flow instabilities by streamline visualization, it was not possible to describe the qualitative characteristics of the flow due to image-quality issues. 

**Figure 6:**
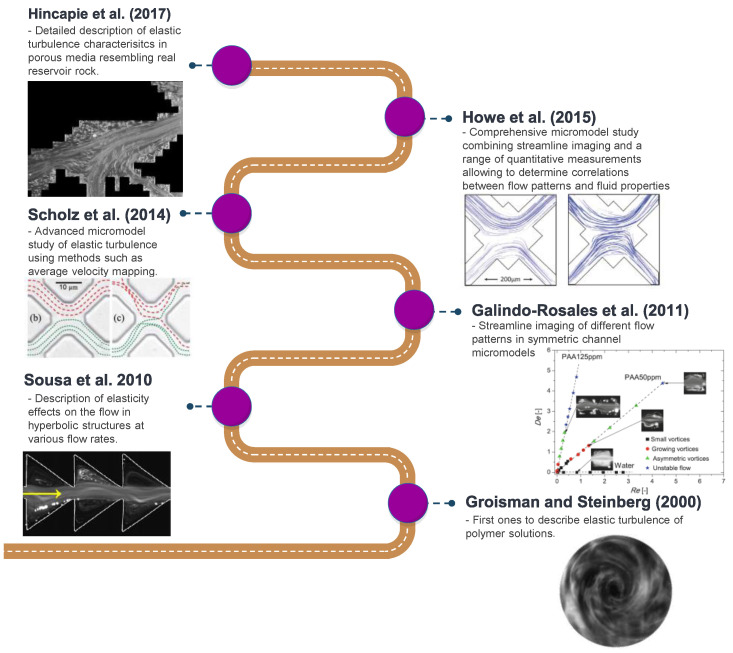
Recent evolution of elastic turbulence visualization (based on Groisman and Steinberg [31], Sousa et al. [73], Galindo-Rosales et al. [61], Scholz et al. [59], Howe et al. [25], Hincapie et al. [29]).

A common problem of these studies is the utilization of symmetric micromodels which are not resembling the complex porous structure of reservoir rocks as encountered in EOR applications. An overview of the recent evolution of streamline imaging during elastic turbulent flow is shown by Figure 6. From this figure it can be clearly seen that all researchers, apart from Hincapie et al. [29], use simplified porous structures which do not represent the complexity of real reservoir rocks.

**Figure 7:**
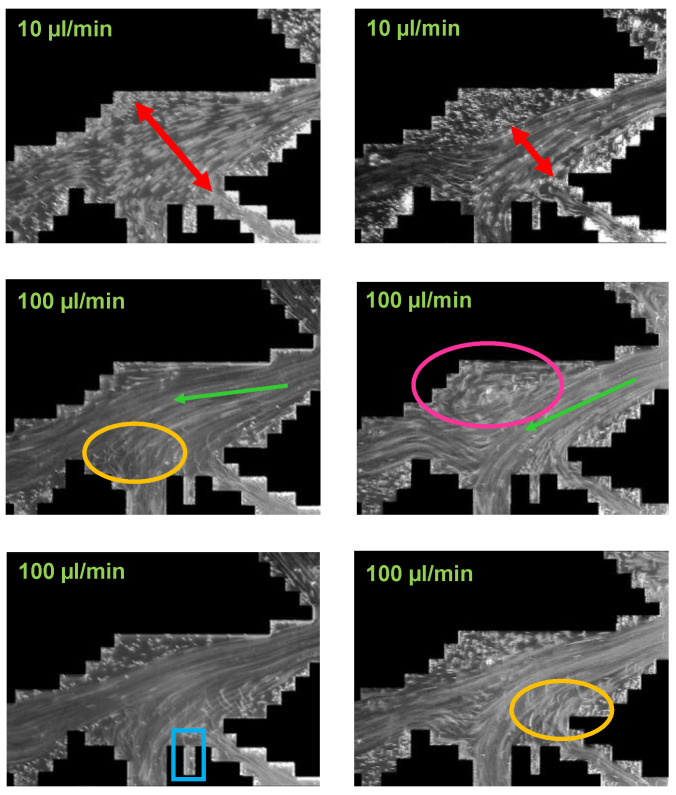
Streamline visualization for a viscoelastic 1000 ppm polymer solution in a glass-silicon-glass (GSG) micromodel (modified after Hincapie et al. [29]).

The work performed by Hincapie et al. [29] accounts for this problem by using glass-silicon-glass (GSG) micromodels generated based on Bentheimer sandstone micro CT images. By this and using state-of-the-art imaging equipment and methods it was possible to describe the elastic turbulence phenomenon of viscoelastic polymer quantitatively and qualitatively in a comprehensive manner. Figure 7 shows the viscoelastic flow instabilities for a 1000 ppm aqueous polymer solution. The images show the streamline visualization for 10 and 100 µL/min in a GSG micromodel. The left and right image in each line have been taken in consecutive time steps of approximately 100 ms. As can be seen, for a flow rate of 10 µL/min there are actually no turbulence characteristics except from a changing flow width of the main stream (indicated by the red arrows) in the pore. Note that this behaviour was only observed for the HPAM solutions here included. In unpublished data from the authors, we have attempted to evaluate polysaccharide solutions (xanthan and others) without similar observations or conditions previously listed. 

Hincapie et al. [29] as well as Rock et al. [30] considered this flow behavior as a transitional flow regime. For 100 µL/min, these researchers observed various elastic turbulent flow characteristics. A remarkable observation is the streamline crossing (indicated by orange circles in Figure 7) which can be described as an overlapping flow of two or more flow levels. The streamline crossing can be observed in particular near grain walls (black) and corners. Another observation from the streamline visualization is the build-up of vortices (indicated by pink circle). By comparison of the left with the right image in the second line, it can be observed that the vortex builds up and disappears within a few tens of ms, clearly illustrating the significant viscoelastic flow instabilities during the flow of the aqueous polymer solution. Another elastic turbulence characteristic that can be defined from Figure 7 is the penetration of the stream into small corners as shown by the light blue rectangle. Again, this penetration is not permanent, but changes every few ms in an irregular manner. A last characteristic that can be observed from the flow visualization is the constantly changing direction of the main stream, which is illustrated by the light green arrows in the bottom images. 

As a summary of the streamline visualization, Hincapie et al. [29] defined the elastic turbulence characteristics as follows: (1) changing stream width, (2) changing stream direction, (3) penetration of the stream into small corners, (4) build-up and immediate collapse of vortices and (5) streamline crossing, especially near grains. Rock et al. [11] consider the penetration of small corners and build-up of vortices, in particular, as the main reason for an increased oil recovery due to viscoelastic effects, as observed by Clarke et al. [55]. This will be discussed in detail in the coming section.

**Figure 8:**
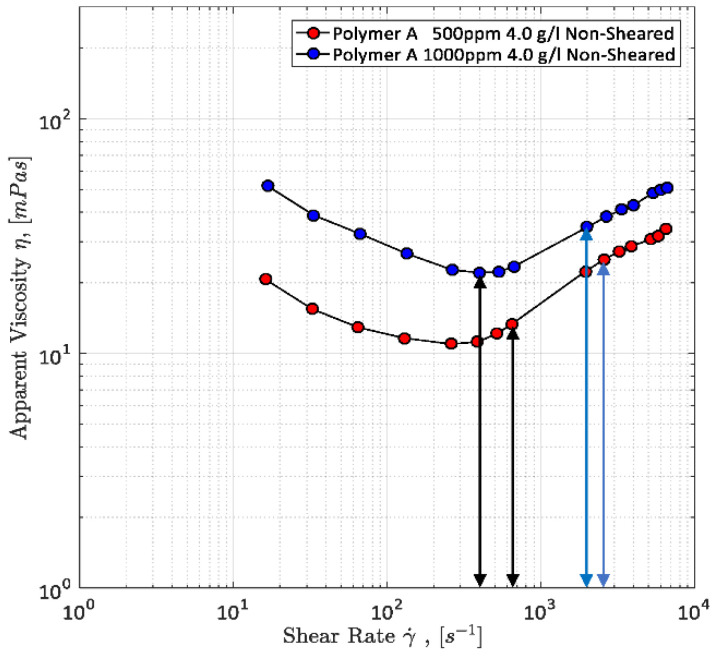
Apparent viscosity vs. shear rate measured in a GSG micromodel. The black lines illustrate the onset of the transitional flow pattern whereas the blue lines illustrate the onset of elastic turbulence (Rock [69]).

In addition to the qualitative analysis of Hincapie et al. [29], Rock [69] conducted in situ micromodel viscosity measurements. An exemplary measurement is shown by Figure 8 which also includes the observed onset of elastic turbulence. As can be seen, the onset of shear thickening correlates with the onset of transitional flow. This underlines the previous assumption in Section 3.2.2 that shear thickening is not an increasing viscosity, but an additional pressure drop along the porous media resulting in a delusory increased viscosity. Howe et al. [25] and Galindo-Rosales et al. [61] have made similar correlations.

### 3.3. Experimental Approaches for the Assessment of Polymer Viscoelasticity

This section provides an overview of experimental investigation methods used to determine the viscoelasticity of polymer solutions. Since many different studies on polymer viscoelasticity have been published over the last two decades, a broad range of experimental approaches, including quantitative and qualitative methods, is available. For the investigation of polymer viscoelasticity with regards to EOR applications, both, the fluid properties itself as well as the flow characteristics in porous media need to be investigated. Therefore, this section is subdivided into fluid property and flow effect parts.

#### 3.3.1. Quantitative Fluid Property Evaluations

Fluid property evaluation usually considers a variety of rheological measurements including viscosity measurements, optical methods and in general chemical and physical properties. Here, only a focus on the viscoelasticity measuring methods is given, since this study focuses on the role of viscoelasticity in enhanced oil recovery applications. 

##### Measurement of Relaxation Time

**Figure 9:**
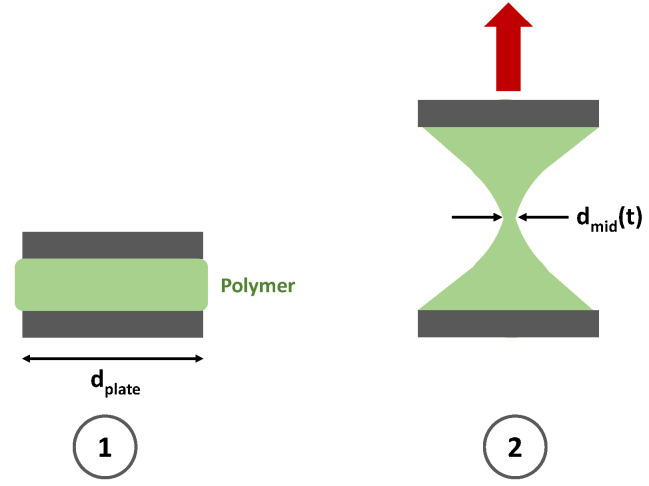
Measurement process of a CaBER™ rheometer. (**1**) Introducing the polymer between plates to form a fluid bridge, (**2**) Rapid movement of the upper plate to create filament.

As already mentioned, the relaxation time λ is one of the most important properties of a polymer solution describing its degree of viscoelasticity. Sousa et al. [73] describe different devices that are capable of measuring the elongational relaxation time of viscoelastic polymer solutions. A suitable rheometer for relaxation time measurements is, for example, the filament stretching extensional rheometer (FiSER) [74,75,76,77]. This rheometer separated the end plates at an exponential rate resulting in a constant deformation rate. Another common device for relaxation time measurements is the capillary break-up extensional rheometer (CaBER™) [27,78,79]. A schematic sketch of the measurement with a CaBER™ can be seen in Figure 9. In a first stage the polymer solution is introduced between two cylindrical plates, usually having a diameter between 4–6 mm. As a result, a fluid bridge between the two plates of the rheometer is formed (see step 1 in Figure 9). Subsequently, the upper plate is rapidly moved upwards and consequently a fluid filament is created and the diameter in the middle is measured over time (see step 2 in Figure 9). The diameter measurement is taken by using a light source and camera [80].

Other approaches of measuring the relaxation time of viscoelastic polymer solutions presented by Sousa et al. [73] are the use of a Rayleigh–Ohnesorge jet elongational rheometer (ROJER) or optically-detected elastocapillary self-thinning dripping-onto-subtract (ODES-DOS). In particular, the ROJER rheometer is capable of measuring relaxation times down to 80 µs. In the work presented by Rock [69], there have been problems with measuring the relaxation times of some 500 ppm HPAM solutions, because the Kinexus rotational rheometer (Malvern Instruments) used in this research was not able to measure the relaxation times. Therefore, a quantitative characterization of these solutions was limited. Here, the ROJER rheometer can solve these problems and hence, should be considered for future relaxation time measurements. Rock [69] measured the elastic (energy storage) modulus G’ and viscous (energy loss) modulus G’’ for various, increasing shear rates. Based on this measurement it was possible to determine the relaxation time of the viscoelastic solution by using,

(13) $$\lambda =\frac{1}{{\dot{\gamma }}_{\mathrm{overlap}}}$$

where *γ*_overlap_ is the shear rate at which the curve of G’ and G’’ overlap in the plot. However, and as mentioned before, this measuring method is only suitable for polymer solutions with relatively high viscoelasticity and consequently, a high relaxation time. 

Recently, Del Giudice et al. [81] presented a microfluidic approach to measure the shear relaxation time of viscoelastic fluids. The measurement principle is based on the flow behavior of solid particles in solution. For a stationary, laminar flow behavior of a Newtonian fluid the particles added to the fluid will move perpendicular to the flow direction in a microfluidic channel. Without inertia and in the constant-viscosity regime of aqueous viscoelastic polymer solutions, the added particles will move to the center line. By this difference in particle flow behavior it is possible to determine the shear relaxation time of diluted polymer solutions. 

**Figure 10:**
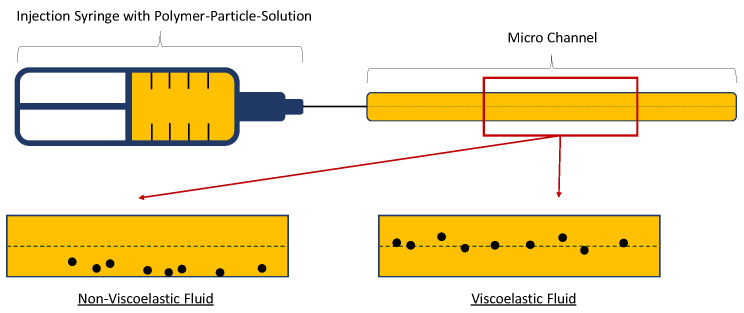
Microfluidic setup for measuring relaxation time of viscoelastic polymer solutions (modified after Del Giudice et al. [81]).

A sketch of a microfluidic setup based on the work of Del Giudice et al. [81] is shown in Figure 10. As it can be seen, the viscoelastic polymer solution with solid particles added to it is injected over a syringe and capillary into a microfluidic channel which has a centerline. In the work presented by Del Giudice et al. [81], the micro channel has a diameter of 100 µm and a length of 7 cm. Using the concept of particle movement discussed before and with the help of a microscope, it is possible to see whether the injected solution is viscoelastic. 

Moreover, by using empirical equations it is possible to calculate the shear relaxation time of the injected polymer solution. First of all, Romeo et al. [82] defined a dimensionless parameter *θ* which correlates *D*e, the length Lmicro and Hmicro of the micro channel as well as the ratio *β* (= Dp/Hmicro whereas Dp is the diameter of the added particles). The dimensionless parameter θ is given by:

(14) $$\theta =De\;\frac{L_{\mathrm{micro}}}{H_{\mathrm{micro}}}\;\beta^{2}=\frac{1}{2}\;\pi \;\lambda \;\frac{4Q}{\pi D^{3}}$$

where *Q* is the injection rate and *D* is the diameter of the channel, since it is cylindrical shaped. As defined previously in Equation (10), a parameter k = ½ *π* is introduced to the definition of *De* in order to transform the calculated relaxation time from s/rad to s. Furthermore, *v*_s_/L was changed to the injection rate with the flow rate *Q*. 

In order to be able to calculate the relaxation time, another equation is required, which is also defined by Romeo et al. [82] and which is:

(15) $$f_{1}=\frac{1}{1+B\;e^{-C\;\theta^{2}}}$$

where *f*_1_ is the fraction of particles in the stream on the center line of the microfluidic channel, *B* and *C* are constants with best fits for *B* = 2.7 and *C* = 2.75. Substituting *θ* in Equation (13) with Equation (14) leads then to the following final relaxation time equation:

(16) $$\lambda =2\pi \;\frac{\pi }{4}\;\frac{1}{\beta^{2}}\;\frac{D^{4}}{LQ}\;\sqrt{\frac{1}{C}\;\ln\left(\frac{f_{1}B}{1-\;f_{1}}\right)}$$

By changing the diameter of the micro channel, it is possible to measure even small values of relaxation time. Potential disadvantages are the empirical origin of the equations, especially for the constants *C* and *B*, the qualitative measurement (based on microscopy) and the missing confidence in the results, although Del Giudice et al. [81] showed a good correlation between measurements with the microfluidic approach and conventional rheometer.

##### Measurement of Shear Viscosity

Although shear viscosity is not a property directly related to the viscoelasticity of aqueous polymer solutions, the shear thinning and thickening regime, and especially its onset, are influenced by the viscoelasticity. Therefore, this part of the section focuses on the viscosity measurement of viscoelastic polymers. 

**Figure 11:**
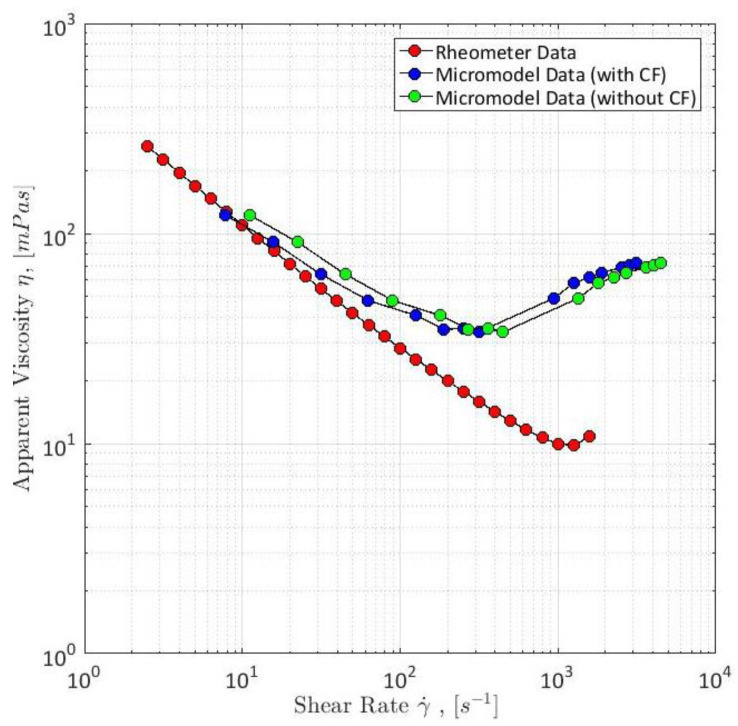
Example for shear rate correction (CF = correction factor) [69].

A simple way of measuring the viscosity of fluids is the use of a rheometer such as a rotational rheometer as used by Hincapie [8]. The results obtained from rheometer are very accurate and reproducible, but do not represent the rheology of fluids within porous media. This was seen in different works, e.g., [8,63,67,83] and Rock [69]. Therefore, it is necessary to measure the rheological properties of the viscoelastic polymer solutions within porous media. Moreover, a combination of both rheometer data and data obtained from flooding experiments within porous media (e.g., sand pack, core plugs and micromodels) allows the correction of the shear rate [60,63,66,72,84]. This is necessary because shear rates are only calculated in flooding experiments and, hence, have a certain degree of inaccuracy. The combination of both data sets and shear rate correction can be seen in Figure 11. 

Another problem of rotational rheometer measurements regarding the evaluation of viscoelastic fluid properties and flow phenomena is the missing shear thickening of the polymers as obtained in experiments in porous media. This can be seen in Figure 11 as well. This further underlines the necessity of experiments in porous media in order to capture the full polymer rheology and, hence, the shear viscosity measurement in porous media is explained below. 

Although core flooding represents the most realistic approach of in situ polymer rheology evaluation, micromodel flooding is here considered to be the best experimental approach due to its setup simplicity, fast measurement and reproducibility of results. Many different microfluidic setups and approaches have been presented or rather proposed such as those from Galindo-Rosales et al. [61], Scholz et al. [59], Howe et al. [25], Clarke et al. [55], Campo-Deano et al. [85], Herbas et al. [86], Wegner et al. [15], Sousa et al. [87], Hincapie [8] and Rock et al. [30]. 

**Figure 12:**
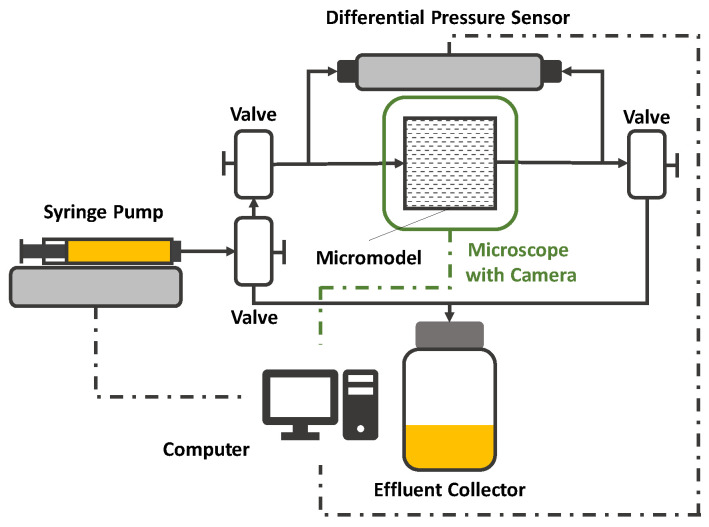
Microfluidic setup for quantitative and qualitative rheological measurements (modified after [69]).

**Figure 13:**
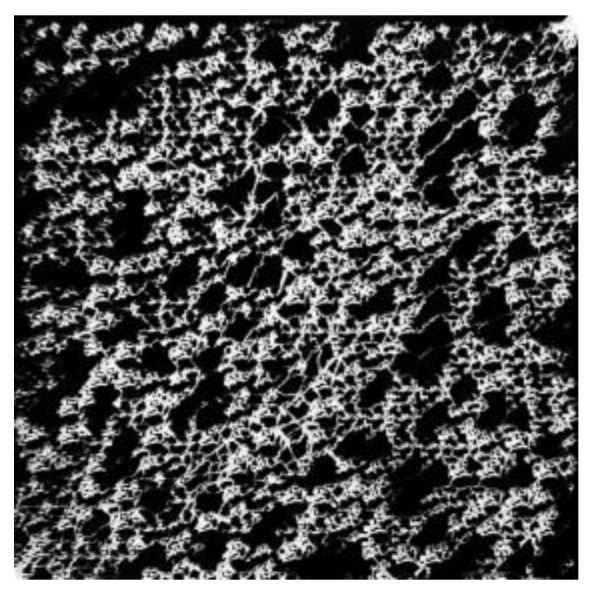
Glass-silicon-glass micromodel (40 × 40 × 0.05 mm) resembling the pore structure of Bentheimer sandstone [30].

In the following, the experimental setup and workflow used for preliminary studies of this work is presented and discussed. The setup presented in Figure 12 consists of a high-precision syringe pump connected to an effluent collector and a differential pressure sensor (quantitative evaluation) and the micromodel by PTFE tubes. All thread connections are additionally isolated by Teflon tape. Furthermore, the micromodel is placed under a microscope equipped with a high-speed camera (qualitative evaluation). There is a broad range of micromodel types that can be used for flooding experiments such as GSG micromodels based on Bentheimer CT images [15,88,89], completely transparent real porous-media-resembling plastic micromodels (Hogeweg et al. 2018) and symmetric micromodels as presented in the work of Galindo-Rosales [61] and Scholz et al. [59]. An overview image of a GSG micromodel as used by Wegner et al. [15], Rock et al. [30], Gaol et al. [89,90] and Hincapie et al. [29] as an example is shown by Figure 13. In addition, valves are installed to allow evacuation of certain parts of the setup, e.g., removing gas bubbles in the pipes. Moreover, the syringe pump, differential pressure sensor, microscope and camera are connected to a computer enabling real-time monitoring of the experiment. The remarkable advantages of this microfluidic setup are the low required fluid volumes, good and fast pressure stabilization, the opportunity of qualitative flow assessment, the reproducibility of results and low number of connections and parts resulting in a reasonably low fraction of failed experiments due to setup failure.

Another advantage of this type of micromodel setup is the easy experimental workflow for the different measurements, from permeability measurements over viscosity evaluations to streamline visualization. The basic polymer evaluation workflow using the setup presented above consists of three steps:

(a) Permeability measurement;
(b) Quantitative polymer characterization including viscosity evaluation; and
(c) A qualitative polymer characterization including streamline visualization.

The latter is explained in detail in the next section. Each experiment starts with a permeability measurement by water flooding. For this, the experimental setup is firstly flooded with CO_2_ in order to guarantee a subsequent trouble-free water saturation of the whole setup. Once the setup is saturated with deionized water, a visual check of the system is performed in order to ensure a gas bubble-free system, because these can cause significant differential pressure fluctuations during the experiment. Subsequently, the micromodel is flooded with deionized water at rates ranging from 100–700 µL/min with flow rate increments of 100 µL/min. Each flooding step is 5 min long to ensure a stabilized differential pressure plateau. Afterwards, the differential pressure is used to calculate the permeability using Darcy’s law. Overall, the average of all 7 flooding steps is used for the final permeability used for subsequent calculations in the quantitative polymer characterization. In addition to the permeability, also the mean grain diameter *D*_p_ has to be determined in order to be able to calculate the Reynolds number given by:

(17) $$Re_{m}=\frac{\delta_{\mathrm{fluid}}\;v_{s}\;D_{p}\;}{\mu_{\mathrm{app}}\left(1-\;\varepsilon \right)}$$

where *δ*_fluid_ is the fluid density, *μ*_app_ the measured, apparent viscosity and *ε* the porosity [91]. Since the sandstone resembling micromodels usually do not consist of single, circle-shaped grains, it is not possible to determine *D*_p_ visually with an image algorithm. Therefore, Rock et al. [11] proposed the rearrangement of the Ergun equation [92] which is given by:

(18) $$D_{p}=\frac{1.75L\delta_{\mathrm{fluid}}\left(1-\varepsilon \right){v_{s}}^{2}+\sqrt{3.0625L^{2}{\delta_{\mathrm{fluid}}}^{2}{\left(1-\varepsilon \right)}^{2}{v_{s}}^{4}+600\mu L{\left(1-\varepsilon \right)}^{2}v_{s}\Delta p\varepsilon^{3}}}{2\Delta p\varepsilon^{3}}$$

where *L* is the flow length along the micromodel, Δ*p* the measured differential pressure along the micromodel and µ the viscosity of the fluid. Since the viscosity of the viscoelastic polymer solutions change with different flow rates, it is necessary to determine *D*_p_ with the pressure data obtained during the permeability flooding with deionized water which is a Newtonian fluid and hence, has no viscosity changes with changing flow rates. 

After permeability and *D*_p_ measurements or rather calculations, the viscosity can be measured. For this purpose, the setup is completely saturated with the polymer solution which has to be rheologically characterized. Subsequently, the polymer is injected at different flow rates ranging from 0.25 µL/min up to 100 µL/min using the syringe pump. The differential pressure along the micromodel is measured each second in order to detect possible fluctuations which indicate problems with the experimental setup. Furthermore, it is highly recommended to use the automated injection function that most of the state-of-the-art syringe pumps offer. By that, an injection program can be set in the pump, so that the injection rate changes every 45 min. This allows the experiment to run overnight. Viscosity is determined by using the measured differential pressure and Darcy’s law. A typical apparent viscosity curve for a viscoelastic polymer solution injected in a GSG micromodel can be seen in Figure 11. The third step of the microfluidic polymer characterization is presented in the next section. The experimental approach presented above is similar to most of the setups found in literature and allows for many different modifications such as the addition of sensors, e.g., flow meter, absolute pressure sensors and gas sensors. 

**Figure 14:**
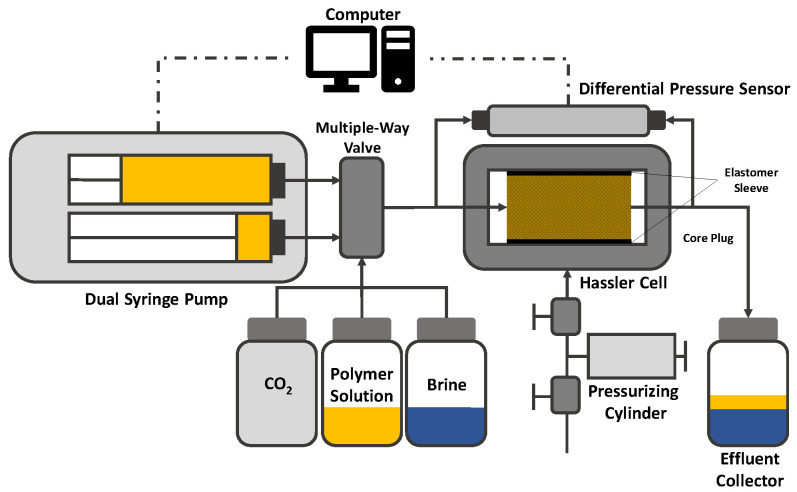
Schematic sketch of a core flooding setup used for experiments at room temperature. For experiments at elevated temperatures the Hassler cell and inlet/outlet pipes are placed in an oven.

The principle of a core flooding setup is basically the same as for the microfluidic flooding setup. The major differences are in the material (steel instead of plastic), the pump (higher injection pressures and volumes are required) and the differential pressure sensor. A schematic sketch of a core flooding setup for apparent viscosity measurements can be seen in Figure 14. Another major difference that can be seen in this setup is the pressurizing cylinder which is necessary in core flooding applications. An elastomer sleeve is put around the core plug, put in the Hassler cell and pressurized manually by adjusting the cylinder outside the cell [93,94,95]. By applying pressure around the sleeve, it is ensured that no injected fluid bypasses the core and, hence, distort, the measurement. As already mentioned in the explanation of the microfluidic setup, various kinds of modification can be added to the setup. The experimental workflow for single-phase polymer flooding using core plugs is basically the same as presented before for the micromodel experiments. 

**Figure 15:**
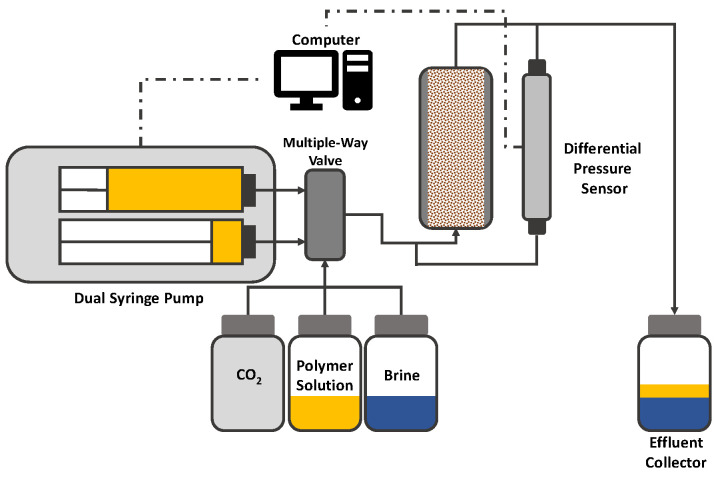
Schematic sketch of a sand pack setup used for experiments at room temperature. For experiments at elevated temperatures, the cell containing the sand and the inlet/outlet pipes needs are placed in an oven.

Another approach for single-phase polymer flooding in porous media is the use of a sand pack as porous media [96,97,98]. A typical sand pack setup can be seen in Figure 15. Instead of a Hassler cell containing a core plug, a cylinder filled with sand is used as porous media. Thereby, the sand has a defined grain diameter which allows for very accurate shear rate calculations; compared to the microfluidic setup where *D*_p_ has to be calculated on the basis of the initial water flooding. Overall, the experimental workflow is the same as described for micromodel flooding.

##### Other Viscoelastic Properties

In addition to the relaxation time and shear viscosity of viscoelastic polymer solutions, the measurement of additional rheological parameters can be used for further fluid characterization which improves understanding of polymer structure and behavior. These rheological parameters include first normal stress difference as elastic and viscous modulus [37,99,100,101]. These are commonly measured by the use of state-of-the-art rotational rheometer, but the moduli, for example, can also be measured using the dynamic light scattering (DLS) technique developed by Duffy et al. [102] and in detail explained by Hincapie [8]. For measurement of extensional viscosity, the small sample viscometer (mVROC™) or the extensional viscometer-rheometer-on-a-chip (eVROC™) as presented by Pipe et al. [103] and Elhajjaji et al. [65] can be used. In this study, based on the results obtained from preliminary experiments prior to this work, it is assumed that the extensional viscosity—often discussed as the additional viscosity in shear thickening—is not the reason for the viscosity increase in porous media seen after the second critical shear rate (see Section 3.2.2 and Section 3.2.3), but the onset of elastic turbulence. This is also supported by the work of Howe et al. [25]. Hence, its measurement is not further discussed in this work. 

#### 3.3.2. Qualitative Flow Evaluation

Further enhancing state-of-the-art optical devices allows the flow of viscoelastic polymer solution in porous media to be characterized qualitatively. This provides additional data in order to understand the viscoelastic phenomena in porous media even better. 

One of the most important qualitative flow evaluations is the streamline visualization during flooding experiments. This is only possible if the porous media is transparent and, therefore, further underlines the advantage of the utilization of micromodels in the evaluation of viscoelastic polymer solutions used in EOR applications. In order to be able to see streamlines during the flow, tracer particles need to be added to the injected solution. The selection of tracer particles is a critical step in the streamline visualization process and, hence, requires a good understanding of the various types of particle offered. A first particle selection criterion is the particle size which is mainly dependent on the resolution and magnification the utilized optical setup offers. Rock et al. [30] and Sousa et al. [87] used microparticles with 1 µm diameter whereas Scholz et al. [59] used microparticles with diameters up to 3 µm. The smallest utilized particles utilized in literature were those of Galindo-Rosales et al. [104] with a diameter of 500 nm. Not only is the particle size a critical selection criterion from a setup point of view, but also from solution stability perspective. Sometimes prepared and well-mixed particle-polymer solutions are stored for later use. Therefore, using smaller particles is beneficial for solution stability since the particle settlement velocity in aqueous fluids is given by Stoke’s law [105]:

(19) $$v_{\mathrm{settlement}}=\frac{1}{18}\;\frac{\left(\delta_{\mathrm{particle}}-\delta_{\mathrm{fluid}}\right)}{\mu_{\mathrm{dynamic}}}\;g\;{D_{\mathrm{particle}}}^{2}$$

where *v*_settlement_ is the particle settlement velocity, *δ*_particle_ the particle density, *δ*_fluid_ the fluid density, *μ*_dynamic_ the dynamic viscosity of the fluid, g the gravitational acceleration and *D*_particle_ the particle diameter. As can be seen from the above equation, the particle diameter influences the velocity with which the particles settle to the bottom of the solution by the power of two. As an example, and assuming equal densities and dynamic viscosity, the particles used by Scholz et al. [59] settle 36 times faster than those used by Galindo-Rosales et al. [104]. It is important to note that the particles can also accumulate at the top of the prepared particle-polymer solution if *δ*_particle_ is smaller than *δ*_fluid_. Another important particle selection criterion is the chemical stability of the tracer particles. Using polymer solutions prepared with deionized water usually does not result in tracer stability problems. In contrast, using high salinity brine for the polymer solutions potentially causes stability problems which result in the formation of agglomerations which will accumulate at the bottom or the top (depending on the particle density) of the solution. Moreover, injection of these instable tracer particle can result in plugging of micromodels. Therefore, knowing the brine composition, salinity and also pH-value of the viscoelastic polymer solution is fundamental for the tracer selection process. In order to withstand harsh polymer solution properties, manufacturers usually offer different kinds of tracer surface modifications such as the addition of NH_2_-, COOH-, NR_3_-, SO_3_H-groups and many others which are not named due to their special use in medical and life science applications [106]. 

Sousa et al. [87] proposed the use of sodium dodecyl sulfate in order to stabilize the tracer particles further in the polymer solution, but this has to be evaluated for polymer solution separately since this can alter the fluid properties. Another selection criterion that has to be considered is the material of the tracer itself, not only due to the chemical interactions with the aqueous polymer solution but also due to the density. By that and regarding Equation (18), it is beneficial to choose tracer particles with a density close to that of the polymer solution in order to guarantee long-term stability. When choosing the tracer particle material, it is also important to consider the imaging method that will be used. If standard light microscopy is used it is recommended to use polystyrene particles as shown in the work of Hincapie et al. [29]. 

Rheological characterizations performed by Hincapie [8] on HPAM solutions showed viscosity alterations of approximately 1% and, thus, polystyrene particles are considered to be suitable for qualitative flow evaluations of viscoelastic polymers. If fluorescence imaging technology is used, it is mandatory to consider the excitation and emission wave length of the particle to ensure that they are visualized by the optical setup. Otherwise, expensive filters and maybe even the light source of the imaging setup needs to be changed. 

Once the tracer particles are chosen and mixed into the aqueous polymer solutions, they can be injected into the transparent micromodels. The calibration of the imaging setup is different from case to case and is therefore not further discussed in this work. Examples of streamline visualizations are shown in Figure 6 and Figure 7. 

Apart from streamline imaging which allows the qualitative description of viscoelastic flow phenomena (e.g., elastic turbulence), the tracer particles can also be used for a quantitative in situ flow characterization by velocimetry [55,59]. 

**Figure 16:**
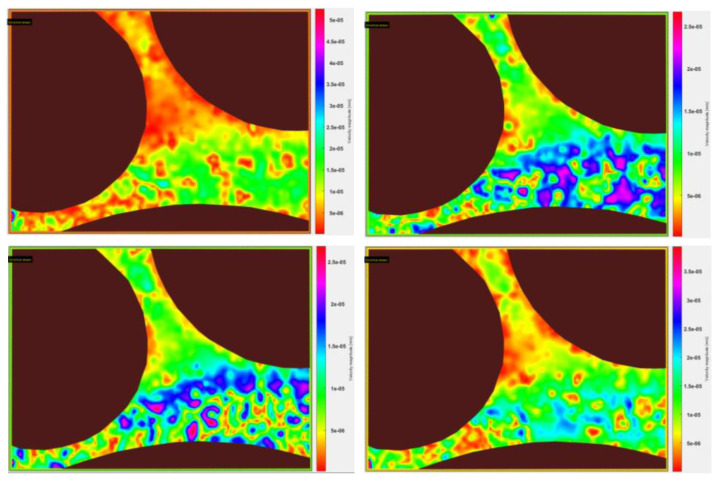
Velocimetry analysis for the flow of a 2000 ppm hydrolyzed polyacrylamide (HPAM) at 1 µL/min flow rate in a porous-media-resembling micromodel performed with PIVlab in MATLAB™. The input images for each velocimetry image were taken with 134 ms time difference.

In velocimetry, stacks of images which were taken in short, consecutive time steps (<100 ms) were used. Thereby, algorithms such as the PIVlab algorithm in MATLAB™ are used to track and analyze the movement of each particle of the flow. By defining the length of one pixel and the time difference between the single images, it is possible for the algorithm to calculate the velocity of each particle and generate an overall velocity map of the flow. An example of such a velocity map from previous experiments is shown in Figure 16. Although a time difference of less than 100 ms between consecutive images is required, 134 ms is acceptable in this case due to the low flow rate of 1 µl/min. Furthermore, it is seen that the scale bar has slightly different scaling which is a problem of the software itself. The results obtained from velocimetry can be used subsequently to calculate other flow properties and also allow for a general analysis of the velocity distribution in the pore space. Moreover, PIVlab offers various kinds of analysis tools as the quantification of vorticity, shear rate, x- and y-direction components of velocity and averages. Additionally, distribution plots can be generated in order to gain an even better overview and understanding of the flow behavior of the polymer in the pore space. A remarkable disadvantage of velocimetry using particle tracing in micromodels is the resolution of tracers, especially at higher flow rates. The higher the flow rate the more the tracer particles tend to appear as a streamline. As a result, the software cannot distinguish the single particles and is not able to perform its analysis on the images. In order to be able to perform velocimetry characterization even at high flow rates, high-speed cameras with more than 500 frames per second are required. Generally, it can be stated that the quality of results increase with an increasing number of frames per second of the camera.

**Figure 17:**
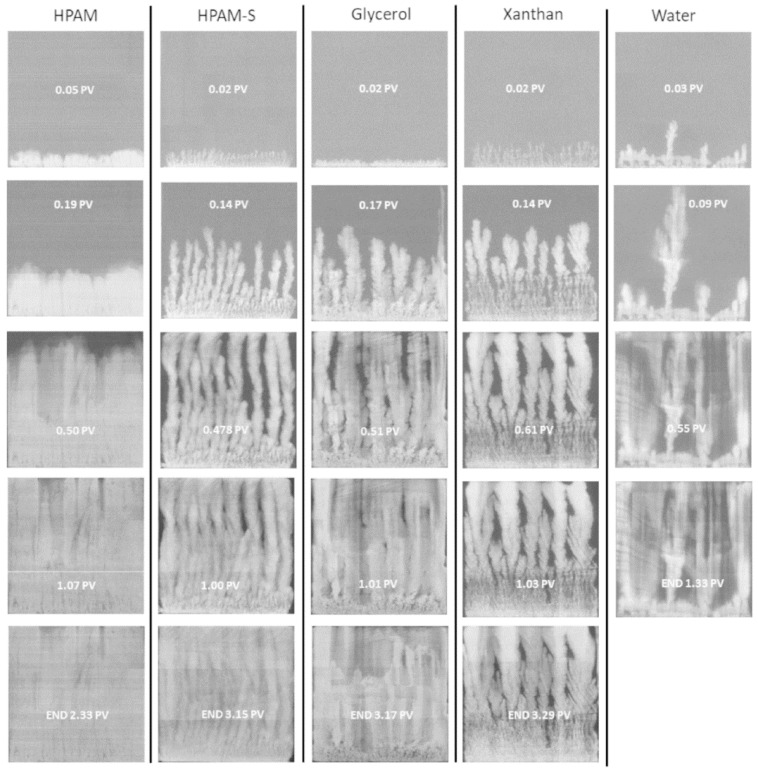
Two-dimensional (2D) X-ray images of oil displacement in sandstone slabs using HPAM, HPAM-S, glycerol, xanthan and water at rates varying between 0.3 and 0.8 mL/min, equaling a typical reservoir rate of 0.3 m/day. [107].

Another qualitative flow evaluation method that was published in literature recently was the use of X-ray imaging on real sandstones. Vik et al. [107] presented this flow imaging approach in detail. X-ray imaging in sandstone samples require a special preparation of the rock. First, the rocks were cut in quadratic slabs which were subsequently coated with epoxy. Afterwards, the sandstone slabs were measured for porosity and permeability, saturated with oil, and aged at 50 °C, over three weeks. Following the ageing process, the sandstone slab was saturated with the oil used in the experiment. Subsequently, the sample was put into a 2D X-ray scanner which had a low-energy source and NaI detector. The researchers state that the minimum time for one X-ray image was approximately 5 min which should not be exceeded in order to visualize the dynamic flow. The result of the flow visualization can be seen in Figure 17. It can be seen that X-ray imaging is capable of visualizing the displacement process in two-phase polymer flooding experiments at a remarkably high resolution. Thereby, this technology provides a significant contribution in the understanding of displacement processes by viscoelastic properties and, hence, it should be considered as a powerful tool in polymer flow characterization. Nevertheless, this visualization method requires expensive equipment which additionally requires well-trained scientific staff to operate it.

### 3.4. Impacts on Polymer Viscoelasticity in Oil Reservoirs

During the preparation, injection and displacement in oil reservoirs, viscoelastic polymer solutions experience a broad range of different potential impacts on their viscoelasticity and thereby on their overall performance in EOR applications. Influencing fluid properties like solvent salinity, polymer concentration and molecular weight alter the in situ rheology of the polymer solution. Furthermore, reservoir rock characteristics and the related mechanical stress have impact as well. Additionally, thermal degradation, chemical alterations and biological degradation by bacteria significantly influence the polymer’s rheology and, therefore, must be studied and understood in order to account for these impacts. Knowledge of potential degradation mechanisms results in an improved polymer optimization and, possibly, results in higher displacement efficiencies. 

#### 3.4.1. Solvent Salinity

A big disadvantage of viscoelastic HPAM polymers is their sensitivity to salinity of the solution which is reflected as a decreased viscosity and relaxation time [10,18,108,109,110,111]. Abidin et al. [18] studied that the main reason behind the decrease of relaxation time and viscosity of HPAM polymers in high-salinity environments are the divalent cations that connect to the acrylate parts of the polymer chains. Moreover, these researchers have shown that divalent cations such as Ca^2+^ and Mg^2+^ have a significantly stronger impact than monovalent cations such as Na^+^ and K^+^. This indicates a correlation between cation type and degree of relaxation time decrease. 

In contrast to this, most of the literature as presented for example in Turkoz et al. [109] assumes that the charge-screening effect is the main driver of relaxation time reduction and therefore, it only depends on salt concentration. The charge-screening mechanism is described by Sasaki [112] as the effect of external charges (cations of the salt) partially bonding to the molecule which are then screened by internal electrons. As a result, the charge of the polymer molecule alters to an electronically neutral state yielding a reduction of the polymer’s viscoelasticity. Although salt concentration is assumed to be the most significant reason for viscoelasticity reduction due to solution salinity, Turkoz et al. [109] investigated the effect of different cation types, e.g., NaCl, C_7_H_5_NaO_3_, KCl, CsCl, CaCl_2_ and ZnCl_2_. 

In contradiction to the previous findings of Abidin et al. [18] claiming a remarkably stronger relaxation time reduction for divalent cations, Turkoz et al. [109] observed no significant differences between monovalent and divalent cations. The authors give a potential explanation for the results obtained by Abidin et al. [18]. During their experiment they observed a distinct pH value decrease for polymer solutions with salts of divalent cations. Therefore, they underline that the results of salinity studies have to be analyzed carefully and critically since pH value alteration is another polymer degradation mechanism. pH-value alteration and its effect on polymer viscoelasticity is discussed in detail later. The researchers assume a correlation between polymer solubility and the Hofmeister series [113] which is defined as follows:

(20) $$Cs^{+}>K^{+}>Na^{+}>Ca^{2+}>Zn^{2+}$$

According to Turkoz et al. [109], the polymer solubility increases to the right of the series and thereby decreases the viscoelasticity. However, the researchers proved that this is only valid for monovalent cations and not for divalent cations. Additionally, they relativize their findings with another correlation they have observed during their rheology measurements. It is seen that the relaxation time alteration can be correlated to the ionic radius of the monovalent cations. Hereby, the larger the ionic radius of the monovalent cations, the stronger is the reduction in the relaxation time of the polymer solution. These researchers have also shown that the ionic radius of the anions has no effect on the polymer’s viscoelasticity. For divalent cations, Turkoz et al. [109] have shown that the hydrated radius of divalent salts has an influence on the relaxation time alteration. Hereby, the effect on the viscoelasticity is more distinctive for larger hydrated radii. 

In conclusion, it can be summarized that with an increasing salinity of the polymer solution and depending on the anion composition of the solution, the viscosity and relaxation time is decreased and, therefore, will have an effect on the polymer flooding efficiency in the oil field. The decrease of viscosity and relaxation time for HPAM polymers have been experimentally shown in preliminary experiments of Rock [69].

**Figure 18:**
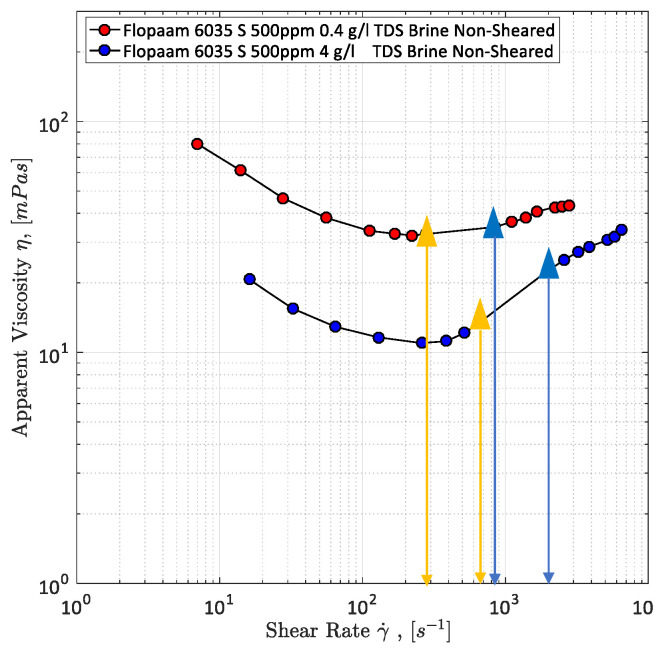
Apparent viscosity measured during micromodel flooding plotted against shear rate. Yellow triangles mark the onset of the transitional flow-regime and blue triangles the onset of elastic turbulence. [69].

Exemplary results are shown in Figure 18. As it can be seen the viscosity of the aqueous polymer solution with 0.4 g/L TDS is significantly higher than the one measured for the solution with 4.0 g/L TDS. Furthermore, the onset for the polymer solution with lower salinity starts at lower shear rates than for the one with higher salinity. This is a strong indication of a stronger viscoelastic response of the low-salinity polymer solution. This result is confirmed by relaxation time measurements performed using a rotational rheometer. The results are shown in Table 2. The relaxation times for the low salinity polymer solution are more than 10 times higher than for the high salinity solution. For the 500 ppm 4 g/L TDS polymer solution it was not possible to measure the relaxation time due to its low value. 

#### 3.4.2. Polymer Concentration

Polymer concentration is the most critical factor influencing the viscoelastic of polymer solutions. Generally, an increase of the polymer concentration results in an increase of viscoelasticity as well as of viscosity. This behavior is proved by a broad range of literature such as the advanced experimental works of Howe et al. [25], Clarke et al. [55], Hincapie [8], Rock et al. [30], Heemskerk [114], Tahir et al. [84] and Seright et al. [115]. 

**Figure 19:**
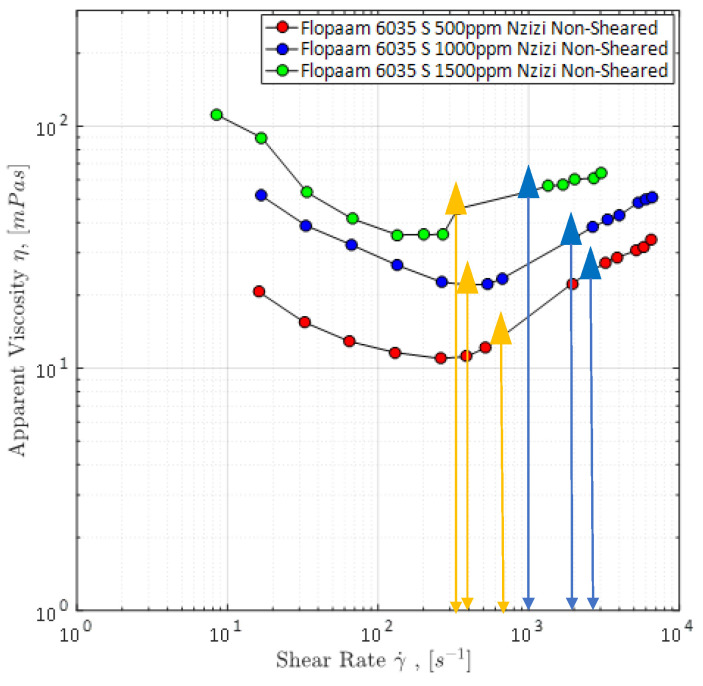
Apparent viscosity of HPAM solutions with different polymer concentrations measured during micromodel single-phase flooding. Yellow triangles mark the onset of transitional flow regime and blue triangles mark the onset of fully developed elastic turbulence [69].

The effect of polymer concentration on the viscoelasticity is illustrated in Figure 19. As can be seen from the plot, the viscosity increases with an increasing polymer concentration. Furthermore, it has be noted that the increase seems to be non-linear. Additionally, the decreasing onset shear rate of the transitional and turbulent flow regime indicate an increasing viscoelasticity of the polymer solutions. This is further underlined by measured relaxation times [69]. In core flooding experiments, an increasing viscoelasticity is observed as well for an increasing polymer concentration [60].

#### 3.4.3. Molecular Weight

The molecular weight Mw of viscoelastic polymer solutions has a large influence on the polymer flood efficiency since its impact on the viscoelasticity is significant. Thereby, using high Mw polymers can significantly enhance the EOR projects’ economics [16].

The impact of Mw on the polymer solutions viscoelasticity is the result of intramolecular effects, especially energetic and steric interactions of chains within the polymer molecule. Specifically, the amino groups of polymers show these interactions which result in an increase of relaxation time as well as of viscosity. Additionally, long polymer chains, hence high Mw polymers, have both a significant advantage and disadvantage regarding the viscoelasticity. The advantage on the one hand is the enhancing degree of polymer chain overlapping resulting in an earlier onset of polymer flow instabilities. On the other hand, it is a remarkable disadvantage that polymer solubility is decreased with an increasing Mw. Therefore, a compromise needs to be defined which again underlines the necessity and benefit of experimental fluid optimization prior to the field application [116]. 

Choosing a viscoelastic polymer with a suitable Mw requires accounting for two main considerations. First, a polymer with the highest possible Mw has to be selected in order to increase or rather maximize the viscoelastic response of the polymer solution and, therefore, reduce the amount of polymer required. Second, the pore size distribution of the reservoir rock needs to be considered in order to ensure a sufficient propagation of the injected polymer solution through the reservoir, otherwise pore plugging can occur and decrease the displacement efficiency. These two considerations can be translated into a simple equation based on empirical evaluations performed on the polymer EOR projects of the Daqing oil field in China. It is given by:

(21) $$M_{w,\max}=11.111\;\left(9.86923*10^{-13}*k_{\mathrm{water}}+0.005\right)$$

where *M*_w, max_ is the maximum *M*_w_ in MDa and *k*_water_ is the water permeability in m^2^. Another correlation that can be used for selection of a proper maximum *M*_w_ is the following ratio:

(22) $$\frac{Average\;Pore\;Throat\;Radius}{Root\;Mean\;Square\;Radius\;of\;Polymer\;Gyration}>5$$

Both guidelines yield to the approximately same result that for reservoirs with an average permeability larger 1000 mD a polymer with a *M*_w_ of 12–16 MDa should be used and a polymer with a *M*_w_ of 17–25 MDa should be selected for permeability larger than 4000 mD. [116].

#### 3.4.4. Mechanical Degradation

Mechanical degradation can be understood as the break-up of polymer chains or rather molecules due to mechanical forces caused by the high shear stress [13,17,43,70,71]. Usually those conditions are encountered in the near-wellbore region and in the equipment such as pumps and valves. The effect decreasing the viscoelastic properties as well as the viscosity of the mechanically degraded polymer solutions is the lower Mw of the polymer chains due to the molecular rupture of them [117]. Furthermore, these researchers showed that the polymer chains break when they are stretched in an entanglement system. This assumption is supported by the work from Bueche [118]. Larsen and Drickamer [119] mention in their work that polymer molecules most likely break at the C–C bonds. 

Bueche [118] discusses the way polymer molecules break up due to mechanical degradation. Firstly, it was assumed that polymer chains break up because of extensive stretching to a point where the stretching force overcomes the bonding force. However, Bueche [118] explains that even at high shear rates the degree of stretching is too low to explain the degradation. A more advanced explanation for the break up is that the polymer rotates significantly fast, so that it is not considerably stretched. As a result, the distortion of the polymer molecule increases which yields to break at a certain point. 

A further mechanism of mechanical degradation explained by Bueche [118] is related to the entanglement of polymer molecules, as already mentioned before. At high shear rates, the degree of entanglement is increased which induce tensional stresses to the molecules in the center of entanglement networks. In addition to the literature presented above, Frenkel [120] found that the probability of breaking is the highest in the center of the polymer molecule. 

In order to avoid any pre-shearing and, therefore, mechanical degradation of the polymer in the surface equipment in the field, Sheng et al. [17] propose to use electromagnetic flowmeters.

#### 3.4.5. Thermal Degradation

A disadvantage of viscoelastic HPAM polymers is their thermal sensitivity [121,122,123]. The acrylamide groups of the polymer start to hydrolyze at 60 °C resulting in sodium acrylate. This yields precipitation and, therefore, a loss of relaxation time and viscosity [124].

This mechanism of thermal polymer degradation is also described by Levitt and Pope [10]. These researchers see the rupture of the acrylic group as the cause of relaxation time and viscosity loss by the reduction of Mw. The reason for the breakdown of the acrylamide groups are free radicals. Regarding the thermal degradation of polymers, carbon-centered radicals are of particular importance and only form when polymerization initiators are thermally decomposed. These radicals are able to abstract hydrogen from the polymer molecule, at least from its backbones. Nevertheless, this process requires oxygen and is therefore strongly limited in the reservoir. 

Levitt and Pope [10] also state that the presence of iron is enough as well for the radicals to hydrolyze the acrylic groups. Another explanation for thermal polymer degradation is given by Maurer and Harvey [121]. These researchers describe thermal degradation in terms of ammonia loss. At elevated temperatures the polymer molecule loses ammonia resulting in the formation of imide which is subsequently decomposed. By that, the polymer loses Mw and finally, viscosity and relaxation time.

The reservoir temperature impact on the viscoelastic behavior of the polymer solution is critical and, therefore, has to be considered in the EOR selection and designing process. Different recommendations for maximum reservoir temperatures are given in Table 3. As it is seen, the highest temperature is the one proposed by Saleh et al. [125], whereas the lowest one is proposed by Saboorian-Joybari [126]. The differences within the literature are the result of the many other criteria which were considered. For example, if a reference recommends a lower temperature, this could be due to a high polymer solution salinity. Therefore, the maximum allowable temperature is reduced to account for the degradation effects of the salinity.

#### 3.4.6. Chemical Degradation

Apart from mechanical and thermal degradation in the reservoir, the polymer also faces chemical degradation [127,128], mainly due to further hydrolysis of its molecules. Hydrolysis of polymers in the reservoir is dependent on the pH-value of the reservoir brine. For smaller polymer molecules, mainly the amide groups are hydrolyzed either by a basic or acidic hydrolysis. Thereby, small molecules are sensitive to low as well as to high pH values. In the case of larger polymer molecules, intermolecular effects can become important, which either accelerate or decelerate the hydrolysis of the polymer in the reservoir. Levitt and Pope [10] state that an acceleration is usually observed at low pH values, whereas high pH values retard the hydrolysis. For this reason, polymers in basic environments usually do not exceed a hydrolysis degree of 66%. The problem of a too high hydrolysis degree is as follows: HPAMs with a hydrolyzed fraction of 33% or higher often encounter chemical stability problems (precipitation) when the solution contains a large number of divalent cations such as Ca^2+^ and Mg^2+^. This issue becomes more severe with an increasing temperature. This perfectly illustrates interactions of different degradation effects/mechanisms [10].

A further problem regarding the chemical degradation of polymers is the oxidation of its molecule by oxygen or rather free radicals, as already explained in Section 3.4.6. Therefore, polymer EOR projects often require the utilization of oxygen scavengers such as hydrazine, sodium bisulfite, sodium hydrosulfite or sulphur dioxide [129].

Overall, chemical degradation resulting from further hydrolysis and oxidation causes a *M*_w_ loss and, therefore, yields to a decreased viscoelasticity. 

#### 3.4.7. Biological Degradation

Biological degradation usually refers to biopolymers such as xanthan gum since these types of polymer are polysaccharides which are a favorable source for bacteria growth. Nevertheless, studies have shown that a small number of sulphate-reducing bacteria (SRB) is able to use viscoelastic HPAM as a source for metabolism [17].

Jia et al. [130] reports that SRB uses HPAM as a source for carbon. Mainly the amide and carbon-containing backbones of the HPAM are used, whereas the amide is an additional source for nitrogen. As a result of the SRBs degrading the HPAM, the polymer chains are broken up resulting in a loss of viscoelasticity by molecular weight reduction. 

Although there is only a very small number of SRBs able to degrade viscoelastic HPAMs, researchers were able to identify them. Jia et al. [130] states that mainly *B. cereus* biologically degrades the HPAM, whereas Al-Moqbali et al. [131] reports *T. mobilis*, *P. aeruginosa* as well as *P. stutzeri* to be the problematic SRBs. Here, it is important to note that Jia et al. [130] mainly refers to offshore polymer flooding projects where sea water can be seen as an additional source for bacteria in the reservoir. This has to be seen as critical since sea water bacteria are aerobic. The bacteria named by Al-Moqbali et al. [131] are aerobic and hence are not further considered to degrade the HPAM in the reservoir. Nevertheless, when mixed in bulk on the surface, biodegradation resulting from these bacteria can occur. 

Apart from reducing the molecular weight and, therefore, viscoelasticity of the polymer solution, SRBs are able to form hydrogen sulfide (H_2_S) resulting in reservoir damage due to souring. Therefore, Sheng et al. [17] recommends the use of biocides such as formaldehyde in order to avoid this kind of biological degradation and for avoidance of reservoir damage.

#### 3.4.8. Impact of Reservoir Rock on the Viscoelastic Flow Behavior 

The impact of reservoir rock has no directly related effect on the viscoelasticity of the polymer solution. However, in the authors’ opinion, it is important to mention the fundamental polymer–solid interactions affecting the overall field application efficiency. 

First of all, it has to be stated that polymer flooding is usually applied to sandstone reservoirs and not to carbonate reservoirs. In the latter case, carbonates have a high ionic exchange capacity and low permeability (in the matrix) and, therefore, the polymer shows a high adsorption or strong plugging respectively [17,132]. 

Considering the effect of the reservoir rock on the polymers’ performance, the most important terms are adsorption and retention. Polymer adsorption means that during the flow of HPAMs the polymer molecules tend to adhere to the surface of the rock. This adsorption takes place until a complete one layer adsorption is reached which results in an irreversible polymer coverage of the reservoir rock. 

Furthermore, adsorption is faster for polymers with higher molecular weight. As a result of polymer adsorption, polymer solution loses 4–30% pore volume (PV). The broad range of polymer loss is the result of varying temperature and salinity [116].

The high polymer losses can not only be explained with adsorption since adsorption happens only until full surface coverage of the rock and there is no polymer-on-polymer adsorption. Hence, retention effects are considered to be responsible for polymer losses in the reservoir. These effects are mainly dependent on the polymer concentration in the injection solution and on brine salinity. Thereby, the polymer retention volume increases with increasing concentration and salinity [116].

The resistance factor RF of the reservoir rock increases during polymer flooding. The resistance factor is generally defined as:

(23) $$RF=\frac{\frac{k_{w}}{{µ}_{w}}}{\frac{k_{p}}{{µ}_{p}}}$$

where *k*_p_ is the relative permeability of the polymer solution and *µ*_p_ the polymer viscosity [13,115]. Seright et al. [13] reported increasing resistance factors for xanthan floods in Berea cores. Assuming constant permeability, only the viscosity affects RF and therefore, an increasing RF is the result of the higher polymer viscosity. More realistically, an increasing resistance factor in reservoir has a two-folded effect: on the one hand, the increasing resistance factor—a synonym for pore plugging—has a beneficial effect in high permeability zones by improving the sweep efficiency. On the other hand, an increased resistance factor means an irreversible permeability reduction which can have a negative effect on subsequent floods. This is referred to as residual resistance factor (RRF):

(24) $$RRF=\frac{k_{2}}{k_{1}}$$

where *k*_2_ is the reservoir brine permeability after and *k*_1_ before the polymer flooding. Thus, an increasing RRF is the result of a permeability reduction. It is important to note that polymers and especially HPAMs lose their ability of pore plugging when undergoing mechanical degradation [133]. For a proper injectivity, Hincapie [69] proposes a RRF below 3.

Kolnes and Nilsson [132] also discuss the rock-related influence on the polymer behavior from a geochemical/lithological perspective. These researchers highly emphasize the value of knowledge of the rock composition. Thereby, three main geochemical or rather lithological factors are important: (1) the presence of CaMg(CO_3_)_2_ and CaCO_3_ (carbonates), (2) FeCO_3_ and (3) clay content. The presence of carbonates strongly controls the pH-value and hence, its viscoelasticity. Furthermore, FeCO_3_ affects the polymer’s stability. The clay content of reservoir rock is important in terms of cationic exchange capacity and by that, it influences the polymer retention. Therefore, the polymer retention increases with an increasing clay content. Apart from the clay fraction in the rock, also the clay type is from a significant importance. Austad et al. [134] report the highest cation exchange capacity for Montmorillonite (80–150 meq/100 g) whereas the lowest is related to kaolinite (3–15 meq/100 g). According to this study, a remarkably higher polymer retention is expected for a sandstone containing montmorillonite rather than kaolinite.

## 4. Viscoelasticity-Related Recovery Mechanisms and Their Importance in EOR Applications

This section focuses specifically on the recovery mechanisms resulting from polymer viscoelasticity. Therefore, mechanisms such as reduction of viscous fingering are not discussed. Sheng et al. [17] state four main recovery mechanisms resulting from polymer viscoelasticity: (1) pulling mechanism, (2) oil-thread flow, (3) stripping mechanism, and (4) shear thickening. 

The pulling mechanism can be understood as a pull-out of oil trapped in dead-end pores. The oil-thread flow becomes especially important after water flooding where the residual oil is divided into single drops. Due to the injection of viscoelastic polymer solutions, the oil drops are pulled together to oil threads. These threads allow the oil to flow towards the residual oil located downstream and thus, results in the formation of an oil bank which can be recovered more easily. 

**Figure 20:**
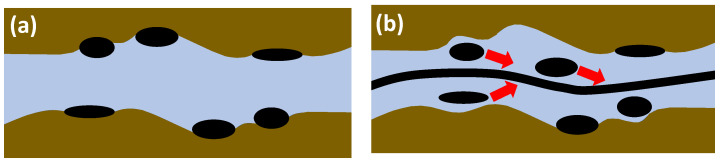
(**a**) Situation after conventional water flooding. The oil is left as isolated oil drops; (**b**) viscoelastic polymer solution is injected resulting in the formation of an oil thread along which the oil upstream flows along and build-up an oil bank downstream.

As can be seen in Figure 20a the oil is left behind as isolated oil drops after water flooding. Due to the injection of viscoelastic polymer solution in Figure 20b, these drops are stretched and thinned resulting in the formation of an oil thread in the pore space. Along this oil thread, the polymer drops flow and form an oil bank downstream which is easier to recover than single oil droplets trapped in the pore structure. Note that the oil thread viewpoint of viscoelastic polymer flooding may be only suitable for explaining the large-sized pores but not small-sized pores or nano-/micro-meter pores. Further research is required is this direction. 

Another mechanism described by Sheng et al. [17] is the stripping mechanism. As the name of the mechanism indicates, the oil on the pore walls is stripped off. The researchers explain this by the higher velocity of the viscoelastic polymer solution at the pore walls. 

The fourth mechanism mentioned by Sheng et al. [17] is the shear thickening which is assumed to increase the viscosity as a result of the viscoelasticity. As already discussed in detail, this study does not assume an actual viscosity increase of the injected polymer, but an additional pressure drop caused by the elastic turbulence. Evidence for this assumption is given by the studies of Hincapie et al. [29] and Rock et al. [11]. These researchers have clearly shown that viscoelasticity is capable of increasing the oil recovery by viscoelastic effects, mainly elastic turbulence. 

**Figure 21:**
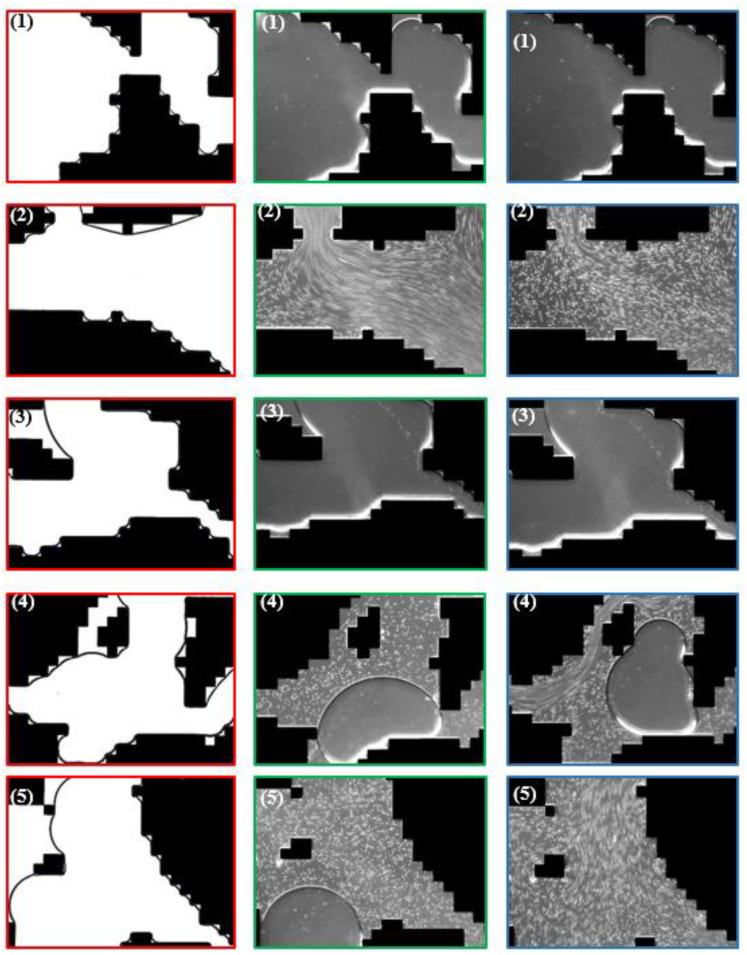
(Red) Binary images of the initial oil saturation in the selected pores. The images refer to the evaluation and analysis of a 500 ppm polymer solution at laminar flow conditions; (green) Streamline visualization during polyethylene oxide (PEO) flooding in the selected pores. The images refer to the evaluation and analysis of PEO (3350 ppm) at laminar flow conditions using an injection rate of 0.5 µL/min; (blue) Streamline visualization during 500 ppm polymer flooding in the selected pores. The images refer to the evaluation and analysis of the 500 ppm polymer solution at laminar flow conditions using an injection rate of 0.5 µL/min [11]. The initial oil saturations (*S*_oi_) for pores 1 to 5 were 97.94%, 95.21%, 90.07%, 86.18% and 84.30%. After *S*_oi_ was determined, non-viscoelastic polyethylene oxide (PEO) were injected (0.5 µL/min) in order to displace the oil without any viscoelastic effects. The residual oil saturations (*S*_or_) after PEO flooding from pores 1 to 5 were 97.94%, 0.00%, 90.07%, 34.64% and 20.18% respectively.

**Figure 22:**
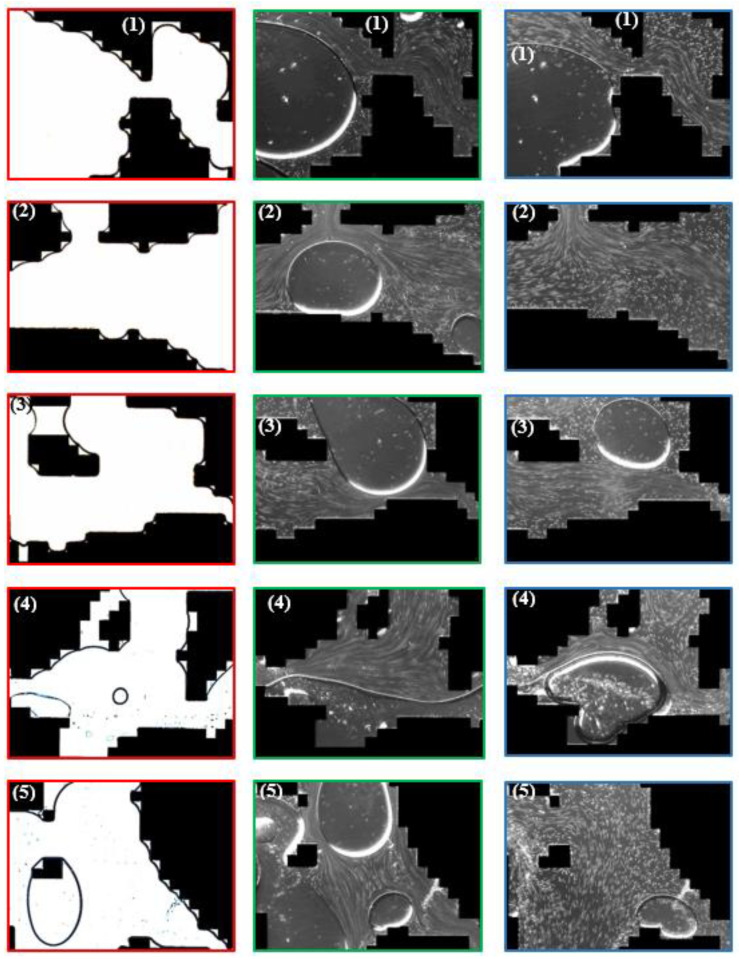
(Red) Binary images of the initial oil saturation in the selected pores. The images refer to the evaluation and analysis of a 500 ppm polymer solution at laminar flow conditions; (green) Streamline visualization during PEO flooding in the selected pores. The images refer to the evaluation and analysis of PEO (3350 ppm) at laminar flow conditions using an injection rate of 0.5 µL/min; (blue) Streamline visualization during 500 ppm polymer flooding in the selected pores. The images refer to the evaluation and analysis of the 500 ppm polymer solution at laminar flow conditions using an injection rate of 0.5 µL/min [11]. The initial oil saturations were determined for pores 1 to 5 to be 93.87%, 96.46%, 90.99%, 87.47% and 74.43%. Similar to the laminar flow regime experiment, a 6500 ppm non-viscoelastic PEO solution was injected (30 µL/min) resulting in *S*_or_ from pores 1 to 5 of 45.38%, 28.07%, 36.39%, 34.7% and 58.69%.

Figure 21 and Figure 22 show the experimental results of Rock et al. [11]. In Figure 21 the results for the laminar flow regime can be seen. On the left side (red) the initial oil saturation is shown which was quantified by using a MATLAB^®^ algorithm. The initial oil saturations (*S*_oi_) for pores 1 to 5 were 97.94%, 95.21%, 90.07%, 86.18% and 84.30%. After *S*_oi_ was determined, non-viscoelastic polyethylene oxide (PEO) were injected (0.5 µL/min) in order to displace the oil without any viscoelastic effects. The residual oil saturations (*S*_or_) after PEO flooding from pores 1 to 5 were 97.94%, 0.00%, 90.07%, 34.64% and 20.18% respectively. Therefore, as can be seen there are some pores where all the oil was recovered and some pores where no oil was displaced. In order to see the influence of viscoelastic recovery mechanisms at a laminar flow regime, a 500 ppm polymer solution which has the same viscosity as the previously injected 3350 ppm PEO solution was injected (0.5 µL/min). *S*_or_ after the flood for pores 1 to 5 had determined to be 97.94%, 0.00%, 90.07%, 37.19% and 0.00%. Overall, the additional recovery was 4.43% due to viscoelastic effects from the injected HPAM. 

In order to determine the recovery efficiency of elastic turbulence, Rock et al. [11] performed the same experiment for a higher flow rate to create elastic turbulence in the porous-media resembling a GSG micromodel. The qualitative results from streamline visualization are seen in Figure 22. The initial oil saturations were determined for pores 1 to 5 to be 93.87%, 96.46%, 90.99%, 87.47% and 74.43%. Similar to the laminar flow regime experiment, a 6500 ppm non-viscoelastic PEO solution was injected (30 µL/min) resulting in *S*_or_ from pores 1 to 5 of 45.38%, 28.07%, 36.39%, 34.7% and 58.69%. Afterwards, the viscoelastic HPAM was injected (30 µL/min) resulting in final *S*_or_ from pores 1 to 5 of 48.08%, 0.00%, 17.49%, 35.30% and 8.35%. This represents an additional oil recovery of 21.95% PV which is significantly higher than at a laminar flow regime without elastic turbulence. Therefore, it can be assumed that the most important viscoelastic recovery mechanism is the elastic turbulence. 

It has to be noted that elastic turbulence is usually observed at shear rates which are several times higher than those encountered in the reservoir. Therefore, although the oil recovery is significantly higher for elastic turbulence, it is not applicable in the field yet. Here, viscoelastic polymer solutions have to be developed which show a remarkable earlier onset of elastic turbulence. The elastic turbulence characteristics responsible for the high additional oil recovery can be seen in Figure 22 and can be summarized as streamline crossing, fluctuation/stream curvature of the main stream and penetration of the stream into small corners of the grains. 

Although the results of this work show promising insights, it has to be seen critically. The shear rates required to see elastic turbulence are significantly higher than observed in the reservoir. In the results presented in Figure 22, the shear rate is approximately 1300 s^−1^, which is about 10 (in the near-wellbore region) to 1000 times (deep in the reservoir) higher than in the reservoir. Therefore, results shown here are not transferable to a realistic field case. 

In contradiction to this study, Howe et al. [25] show an onset of elastic turbulence for a shear rate of approximately 1–10 s^−1^ for core flooding. These rates allow for the assumption that viscoelastic oil recovery can take place at typical reservoir rates. Nevertheless, these experiments are single phase. Finally, and as a result of this study, the on-going question of this work must be: when is elastic turbulence onset really observed under reservoir conditions and in two- or even three-phase flow? If it is encountered at reservoir shear rates, viscoelasticity plays a significant role in polymer EOR, if it is not, the role of polymer viscoelasticity is strongly reduced. 

A general overview and summary of viscoelasticity’s role in EOR applications is given in the next sections.

## 5. Polymer Viscoelasticity and Enhanced Oil Recovery: A Two-Folded Partnership

As seen before, polymer viscoelasticity can have a significant impact on the polymer flooding efficiency resulting in additional oil recovered from mature fields. Assuming shear thickening not to be a viscosity increase, mainly the elastic turbulence flow characteristics presented in Section 3.2.3 are responsible for an incremental recovery due to viscoelasticity. 

**Figure 23:**
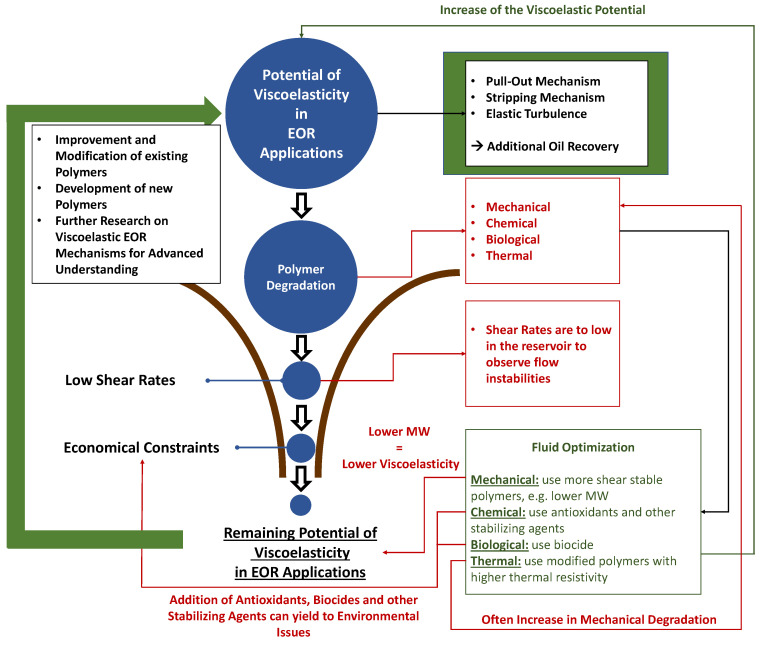
Potential and impacts on the role of polymer viscoelasticity in EOR applications. In contrast to laboratory studies, a decreasing potential is observed taking all impacts and interactions into account. Modification of existing and development of new polymers in combination with further research can drastically improve the viscoelastic influence in polymer flooding.

Although the results presented and discussed in literature are promising, the realistic situation appears to be different as can be seen from Figure 23. When neglecting all the impacts on polymer viscoelasticity, the potential of viscoelasticity in EOR applications is remarkable. But first a decrease of viscoelasticity’s potential has to be made when taking polymer degradation into account. Mechanical, chemical, biological and thermal degradation result in a lower relaxation time of the polymer. Hence, the viscoelastic recovery mechanisms discussed in the section before are weaker. A second and actually an even more significant decrease of viscoelasticity’s importance in EOR is the result of the low shear rates observed in the reservoir. 

Typically, shear rates of 1 (deep in the reservoir) to 100 s^−1^ (near-wellbore region) are achieved. This is too low to see flow instabilities which have been shown to be the main viscoelastic recovery mechanism. In order to account for these drastic impacts, the injected polymer solution can be optimized. Nevertheless, fluid optimization with regards to chemical and biological resistance include the utilization of chemical agents and biocides which can cause environmental issues.

In order to achieve promising results from experimental research in field applications, it is absolutely necessary to improve or rather modify existing polymers. This can yield polymers that are more resistant to the various kinds of degradation and, therefore, have a higher viscoelasticity. In addition, the modification of polymer can result in polymers that show elastic turbulent flow at lower shear rates and, hence, may become applicable in reservoirs. Not only the modification of existing but also the development of new polymer molecular structures can yield this result. 

Most importantly, viscoelastic EOR mechanisms need to be further extensively investigated and discussed in order to understand the importance of the single mechanisms. The work presented in this study indicates that elastic turbulence is the main mechanism behind viscoelastic oil recovery. Therefore, an advanced understanding of elastic turbulence, its characteristics, its triggering effects as well as its contribution to oil recovery is absolutely necessary. 

## 6. Summary and Conclusions

In this work the role of polymer viscoelasticity was discussed in detail. A focus was given to the chemistry of viscoelastic polymers (especially HPAMs), to the physical and mathematical description of viscoelasticity itself, as well as to the viscoelastic flow phenomena including shear thinning, shear thickening and elastic turbulence. Furthermore, this study gave an extensive overview of the experimental approaches used in current and well-established literature. Experiments for quantitative and qualitative evaluation of polymer viscoelasticity were presented. Additionally, this work gave an in-depth overview of the different impacts on the polymer’s viscoelasticity in EOR applications as well as of the different types of degradation a polymer encounters in the reservoir. Finally, this study extensively discusses the viscoelasticity-related oil-recovery mechanisms underlining the significant importance of elastic turbulence.

Structurally speaking, the chemistry of viscoelastic polymers such as HPAM can strongly vary and allows a large number of molecule modifications. Strong impacts on viscoelasticity are observed from high salinities, mechanical, thermal, chemical and biological degradation. EOR polymer solutions require the performance of fluid optimization in terms of concentration and molecular weight.

Viscoelastic flow phenomena encountered in porous media, e.g., sandstones, are shear thinning, shear thickening and have elastic turbulence, whereas shear thickening is not a viscosity increase, but an additional pressure drop. Viscoelasticity-related recovery mechanisms can be summarized as a pulling and stripping mechanism, oil-thread flow and elastic turbulence.

Regarding the dominance of mechanisms, shear thickening is not a viscosity increase as indicated by the apparent viscosity measurements, but an additional pressure drop caused by elastic turbulence. Elastic turbulence is assumed to have the most significant effect on additional oil recovery as was shown by two-phase experiments. This is summarized as changing stream width, changing stream direction, and penetration of the stream into small corners, build-up and immediate collapse of vortices and streamline crossing. 

Overall, we present a good justification for the potential or limits of polymer viscoelasticity on oil recovery. The industry is highly divided on the interpretations of the data concerning viscoelasticity. From the operational point of view these appear challenging and unclear, whereas they are much easier to digest from the laboratory side. Further studies continue suggesting mixed data on the effect on oil recovery or the reduction on residual oil saturation due to the use of polymers.

## Figures and Tables

**Table 1:** Overview of the mechanism reported that could explain polymer enhanced oil recovery (EOR) applications.

	Mechanism	Reference
1	Relative Permeability Reduction	Sheng et al. [17], Sheng [14], Huh and Pope [9], Abidin et al. [18]
2	Viscous Fingering Reduction	Sheng et al. [17], Sheng [14], Clarke et al. [2], Dong et al. [16], Abdul Hamid and Muggeridge [19], Tahir et al. [20]
3	Enhanced Flow between Vertical, Heterogeneous Layers	Sheng et al. [17], Sorbie [21]
4	Pull-Out in Dead End Pores	Luo et al. [22], Yin et al. [23]
5	Stripping from Oil-Wet Rock Surface	Wang [24]
6	Shear Thickening	Rock et al. [11], Hincapie [8], Howe et al. [25], Clarke et al. [2], Azad and Trivedi [26,27,28]
7	Elastic Turbulence	Hincapie et al. [29], Rock et al. [30], Groisman and Steinberg [31]

**Table 2:** Relaxation times of HPAM polymers with different solution salinity and polymer concentrations [69].

Polymer Concentration, c [ppm]	Solution Salinity [g/l TDS]	Molecular Weight, MW [MDa]	Relaxation Time, λ [s]
500	4.0	29	n.a.
1000	4.0	29	2.31
1500	4.0	29	3.33
500	0.4	29	17.22
1000	0.4	29	25.62
1500	0.4	29	40.93

**Table 3:** Literature recommended maximum reservoir temperatures for polymer flooding in oil reservoirs (modified after [35]).

Reference	Recommended Maximum Reservoir Temperature [°C]
Taber et al. (1997)	<93
Saleh et al. (2014)	<99
Dickson et al. (2010)	<77
Delamaide et al. (2016)	<80
Saboorian-Joybari (2015)	<65

## Abbreviations and Symbols

AMPS	2-acrylamide-2-methyl
CaBER	Capillary break-up extensional rheometer
CT	Computer tomography
DLS	Dynamic light scattering
EOR	Enhanced oil recovery
eVROC	Extensional viscometer-rheometer-on-a-chip
FiSER	Filament stretching extensional rheometer
GSG	Glass-Silicon-Glass
HPAM	Hydrolyzed polyacrylamide
ODES-DOS	Optically-detected elastocapillary self-thinning dripping-onto-substract
PAM	Polyacrylamide
PEO	Polyethylene oxide
PTFE	Polytetrafluorethane
PV	Pore volume
RF	Recovery factor
ROJER	Rayleigh-Ohnesorge Jet Elongational Rheometer
SRB	Sulphate-reducing bacteria
B	Deformation tensor [-]
γ	Shear rate [s^−1^]
*γ*_overlap_	Overlap shear rate G’ vs. G’’ [s^−1^]
*D*_e_	Deborah number [-]
*D*_p_	Mean grain diameter [m]
*D*_particle_	Particle diameter [m]
*δ*_fluid_	Fluid density [kg/m³]
*δ*_particle_	Particle density [kg/m³]
Δp	Differential pressure [Pa]
ε	Porosity [-]
*f*_1_	Particle fraction [-]
*φ*_1,2_	Time derivates [-]
g	Gravitational acceleration [m/s²]
G	Relaxation modulus [Pa.s]
G’	Storage modulus [Pa.s]
G’’	Loss modulus [Pa.s]
*H*_micro_	Microchannel height [m]
*k*_water_	Relative water permeability [m²]
L	Flow length [m]
*L*_micro_	Microchannel length [m]
λ	Relaxation time [s]
*M*_w_	Molecular weight [MDa]
*M*_w, max_	Maximum molecular weight [MDa]
µ	Viscosity [Pa.s]
*µ*_app_	Apparent viscosity [Pa.s]
*µ*_dynamic_	Dynamic viscosity [Pa.s]
*N*_1,2_	Normal stress difference [Pa]
η	Shear viscosity [Pa.s]
Q	Flow rate [m³/s]
*Re*_m_	Modified Reynolds number [-]
*S*_oi_	Initial oil saturation [-]
*S*_or_	Residual oil saturation [-]
*σ*_xx_	Normal stress in x-direction [Pa]
*σ*_yy_	Normal stress in y-direction [Pa]
t	Time [s]
τ	Shear stress [Pa]
*v*_s_	Superficial velocity [m/s]
*v*_settlement_	Particle settlement velocity [m/s]
ν	Elastic stress [Pa]
*W*_i_	Weissenberg number [-]

## References

[1] Wylde J.J., Slayer J.L., Barbu V. Polymeric and Alkali-Surfactant Polymer Enhanced Oil Recovery Chemical Treatment: Chemistry and Strategies Required After Breakthrough into the Process. Proceedings of the SPE International Symposium on Oilfield Chemistry.

[2] Clarke A., Howe A.M., Mitchell J., Staniland J., Hawkes L.A. How Viscoelastic Polymer Flooding Enhances Displacement Efficiency. Proceedings of the SPE Asia Pacific Enhanced Oil Recovery Conference.

[3] Hon V.Y., Kechut N.I., Razak W.N.A.W. Enhanced Oil Recovery Potential of Heavy-Oil Fields in Africa. Proceedings of the International Oil Conference and Exhibition in Mexico.

[4] Iwere F.O., Heim R.N., Cherian B.V. Numerical Simulation of Enhanced Oil Recovery in the Middle Bakken and Upper Three Forks Tight Oil Reservoirs of the Williston Basin. Proceedings of the SPE Americas Unconventional Resources Conference.

[5] Wu Y., Ma D., Liu S., Wang H., Zhao X. EOR of Superheated Steam Injection in Shallow Heavy Oil Reservoir: A Case Study. Proceedings of the International Oil and Gas Conference and Exhibition in China.

[6] Sahin S., Kalfa U., Celebioglu D. Unique CO_2_-Injection Experience in the Bati Raman Field May Lead to a Proposal of EOR/Sequestration CO_2_ Network in the Middle East. Proceedings of the SPE International Conference on CO_2_ Capture, Storage, and Utilization.

[7] Zhang Y.P., Huang S., Dong M. (2005). Determining the Most Profitable ASP Flood Strategy for Enhanced Oil Recovery. J. Can. Pet. Technol..

[8] Hincapie R.E. (2016). Pore-Scale Investigation of the Viscoelastic Phenomenon during Enhanced Oil Recovery (EOR) Polymer Flooding through Porous Media.

[9] Huh C., Pope G.A. Residual Oil Saturation from Polymer Floods: Laboratory Measurements and Theoretical Interpretation. Proceedings of the SPE Symposium on Improved Oil Recovery.

[10] Levitt D., Pope G.A. Selection and Screening of Polymers for Enhanced-Oil Recovery. Proceedings of the he SPE Symposium on Improved Oil Recovery.

[11] Rock A., Hincapie R.E., Wegner J., Födisch H., Ganzer L. Pore-scale Visualization of Oil Recovery by Viscoelastic Flow Instabilities during Polymer EOR. Proceedings of the IOR 2017—19th European Symposium on Improved Oil Recovery.

[12] Seright R.S., Wang D., Lerner N., Nguyen A., Sabid J., Tochor R. Beneficial Relative Permeabilities for Polymer Flooding. Proceedings of the SPE Improved Oil Recovery Conference.

[13] Seright R.S., Seheult J.M., Talashek T. Injectivity Characteristics of EOR Polymers. Proceedings of the SPE Annual Technical Conference and Exhibition.

[14] Sheng J.J. (2011). Modern Chemical Enhanced Oil Recovery.

[15] Wegner J., Hincapie R.E., Födisch H., Ganzer L. Novel Visualisation of Chemical EOR Flooding Using a Lab-on-a-Chip Setup Supported by an Extensive Rheological Characterisation. Proceedings of the SPE Asia Pacific Enhanced Oil Recovery Conference.

[16] Dong H., Fang S., Wang D., Wang J., Liu Z.L., Hou W. Review of Practical Experience & Management by Polymer Flooding at Daqing. Proceedings of the SPE Symposium on Improved Oil Recovery.

[17] Sheng J.J., Leonhardt B., Azri N. (2015). Status of Polymer-Flooding Technology. J. Can. Pet. Technol..

[18] Abidin A.Z., Puspasari T., Nugroho W.A. (2012). Polymers for Enhanced Oil Recovery Technology. Procedia Chem..

[19] Hamid S.A.A., Muggeridge A. Viscous Fingering in Reservoirs with Long Aspect Ratios. Proceedings of the SPE Improved Oil Recovery Conference.

[20] Tahir M., Hincapie R.E., Ganzer L. (2020). An Elongational and Shear Evaluation of Polymer Viscoelasticity during Flow in Porous Media. Appl. Sci..

[21] Sorbie K.S. (1991). Polymer-Improved Oil Recovery.

[22] Luo H., Al-Shalabi E.W., Delshad M., Panthi K., Sepehrnoori K. (2016). A Robust Geochemical Simulator to Model Improved-Oil-Recovery Methods. SPE J..

[23] Yin H., Wang D., Zhong H. Study on Flow Behavoirs of Viscoelastic Polymer Solution in Micropore with Dead End. Proceedings of the SPE Annual Technical Conference and Exhibition.

[24] Wang D., Cheng J., Yang Q., Wenchao G., Qun L., Chen F. Viscous-Elastic Polymer Can Increase Microscale Displacement Efficiency in Cores. Proceedings of the SPE Annual Technical Conference and Exhibition.

[25] Howe A.M., Clarke A., Giernalczyk D. (2015). Flow of concentrated viscoelastic polymer solutions in porous media: Effect of MW and concentration on elastic turbulence onset in various geometries. Soft Matter.

[26] Azad M.S., Trivedi J.J. (2019). Quantification of the Viscoelastic Effects during Polymer Flooding: A Critical Review. SPE J..

[27] Azad M.S., Trivedi J.J. (2020). Extensional Effects during Viscoelastic Polymer Flooding: Understanding Unresolved Challenges. SPE J..

[28] Azad M.S., Trivedi J.J. Does Polymer’s Viscoelasticity Influence Heavy Oil Sweep Efficiency and Injectivity at 1ft/Day? In Proceedings of the SPE International Heavy Oil Conference and Exhibition, Kuwait City, Kuwait, 10–12 December 2018.

[29] Hincapie R.E., Rock A., Wegner J., Ganzer L. Oil Mobilization by Viscoelastic Flow Instabilities Effects during Polymer EOR: A Pore-Scale Visualization Approach. Proceedings of the SPE Latin America and Caribbean Petroleum Engineering Conference.

[30] Rock A., Hincapie R.E., Wegner J., Ganzer L. Advanced Flow Behavior Characterization of Enhanced Oil Recovery Polymers using Glass-Silicon-Glass Micromodels that Resemble Porous Media. Proceedings of the SPE Europec featured at 79th EAGE Conference and Exhibition.

[31] Groisman A., Steinberg V. Elastic Turbulence in a Polymer Solution Flow | Nature. https://www.nature.com/articles/35011019.

[32] Chen P., Balasubramanian S., Bose S., Alzahabi A., Thakur G. An Integrated Workflow of IOR/EOR Assessment in Oil Reservoirs. Proceedings of the Offshore Technology Conference.

[33] Moreno J.E., Flew S., Gurpinar O., Liu Y., Gossuin J. Effective Use of Laboratory Measurements on Eor Planning. Proceedings of the Offshore Technology Conference.

[34] Su S., Giddins M.A., Naccache P., Clarke A., Howe A.M. Accurate Modeling of Polymer Enhanced Oil Recovery Corefloods by Reservoir Simulation. Proceedings of the SPE Reservoir Characterisation and Simulation Conference and Exhibition.

[35] Delamaide E. Exploring the Upper Limit of Oil Viscosity for Polymer Flood in Heavy Oil. Proceedings of the SPE Improved Oil Recovery Conference.

[36] Pu W., Jiang F., He Y., Wei B., Tang Y. (2016). Synthesis of a novel comb micro-block hydrophobically associating copolymer for Ca^2+^/Mg^2+^ resistance. RSC Adv..

[37] Cai S., He X., Liu K., Rodrigues A.M., Zhang R. (2017). Macromolecular interactions and synergy in xanthan/HPAM aqueous solutions. RSC Adv..

[38] Menter P. (2000). Acrylamide Polymerization—A Practical Approach. Bio-Rad Tech Note.

[39] Lentz R.D., Andrawes F.F., Barvenik F.W., Koehn A.C. (2008). Acrylamide Monomer Leaching from Polyacrylamide-Treated Irrigation Furrows. J. Environ. Qual..

[40] Hotta M., Kennedy J., Higginbotham C., Morris N. (2016). Durum Wheat Seed Germination Response to Hydrogel Coatings and Moisture under Drought Stress. Am. J. Agric. Biol. Sci..

[41] Zweigle M.L., Lamphere J.C. (1979). Cross-Linked, Water-Swellable Polymer Microgels. U.S. Patent.

[42] Karsani K.S.M.E., Al-Muntasheri G.A., Sultan A.S., Hussein I.A. (2014). Impact of salts on polyacrylamide hydrolysis and gelation: New insights. J. Appl. Polym. Sci..

[43] Divers T., Gaillard N., Bataille S., Thomas A., Favéro C. Successful Polymer Selection for CEOR: Brine Hardness and Mechanical Degradation Considerations. Proceedings of the SPE Oil and Gas India Conference and Exhibition.

[44] Gaillard N., Giovannetti B., Leblanc T., Thomas A., Braun O., Favero C. Selection of Customized Polymers to Enhance Oil Recovery from High Temperature Reservoirs. Proceedings of the SPE Latin American and Caribbean Petroleum Engineering Conference.

[45] The Polyacrylamide Matrix | National Diagnostics. https://www.nationaldiagnostics.com/electrophoresis/article/polyacrylamide-matrix.

[46] Zhu D., Zhang J., Han Y., Wang H., Feng Y. Laboratory Study on the Potential EOR Use of HPAM/VES Hybrid in High-Temperature and High-Salinity Oil Reservoirs. https://www.hindawi.com/journals/jchem/2013/927519/.

[47] Kulicke W., Hörl H. (1985). Preparation and characterization of a series of poly(acrylamide-co-acrylates), with a copolymer composition between 0–96.3 mol-% acrylate units with the same degree and distribution of polymerization. Colloid Polym. Sci..

[48] Spildo K., Sæ E.I.Ø. (2015). Effect of Charge Distribution on the Viscosity and Viscoelastic Properties of Partially Hydrolyzed Polyacrylamide. Energy Fuels.

[49] Meng L., Kang W., Zhou Y., Wang Z., Liu S., Bai B. (2008). Viscoelastic rheological property of different types of polymer solutions for enhanced oil recovery. J. Cent. South Univ. Technol..

[50] Leonhardt B., Ernst B., Reimann S., Steigerwald A., Lehr F. Field Testing The Polysaccharide Schizophyllan: Results of The First Year. Proceedings of the SPE Improved Oil Recovery Symposium.

[51] Needham R.B., Doe P.H. (1987). Polymer Flooding Review. J. Pet. Technol..

[52] Hincapie R.E., Ganzer L. Assessment of Polymer Injectivity with Regards to Viscoelasticity: Lab Evaluations towards Better Field Operations. Proceedings of the EUROPEC.

[53] Mezger T.G. (2012). Das Rheologie Handbuch: Für Anwender von Rotations- und Oszillations-Rheometern.

[54] Macosko C.W. Rheology: Principles, Measurements, and Applications | Wiley. https://www.wiley.com/en-us/Rheology%3A+Principles%2C+Measurements%2C+and+Applications-p-9780471185758.

[55] Clarke A., Howe A.M., Mitchell J., Staniland J., Hawkes L., Leeper K. (2015). Mechanism of anomalously increased oil displacement with aqueous viscoelastic polymer solutions. Soft Matter.

[56] Tahir M. (2020). Experimental Investigation of Sulfate-Modified Water and Polymer Flooding for Enhanced Oil Recovery. Ph.D. Thesis.

[57] Tahir M., Hincapie R.E., Ganzer L. (2020). Influence of Sulfate Ions on the Combined Application of Modified Water and Polymer Flooding—Rheology and Oil Recovery. Energies.

[58] Hincapie R.E., Duffy J., O’Grady C., Ganzer L. An Approach to Determine Polymer Viscoelasticity Under Flow Through Porous Media by Combining Complementary Rheological Techniques. Proceedings of the SPE Asia Pacific Enhanced Oil Recovery Conference.

[59] Scholz C., Wirner F., Gomez-Solano J.R., Bechinger C. (2014). Enhanced dispersion by elastic turbulence in porous media. EPL Europhys. Lett..

[60] Be M., Hincapie R.E., Rock A., Gaol C.L., Tahir M., Ganzer L. Comprehensive Evaluation of the EOR Polymer Viscoelastic Phenomenon at Low Reynolds Number. Proceedings of the SPE Europec featured at 79th EAGE Conference and Exhibition.

[61] Galindo-Rosales F.J., Campo-Deaño L., Pinho F.T., van Bokhorst E., Hamersma P.J., Oliveira M.S.N., Alves M.A. (2012). Microfluidic systems for the analysis of viscoelastic fluid flow phenomena in porous media. Microfluid. Nanofluidics.

[62] Sochi T. (2010). Non-Newtonian flow in porous media. Polymer.

[63] Tahir M., Hincapie R.E., Be M., Ganzer L. (2017). A Comprehensive Combination of Apparent and Shear Viscoelastic Data during Polymer Flooding for EOR Evaluations. World J. Eng. Technol..

[64] Spaull A.J.B., Barnes H.A., Hutton J.F., Walters K. (1989). An Introduction to Rheology.

[65] Elhajjaji R.R., Hincapie R.E., Tahir M., Rock A., Wegner J., Ganzer L. Systematic Study of Viscoelastic Properties During Polymer-Surfactant Flooding in Porous Media. Proceedings of the SPE Russian Petroleum Technology Conference and Exhibition.

[66] Tahir M., Hincapie R.E., Be M., Ganzer L. Experimental Evaluation of Polymer Viscoelasticity During Flow in Porous Media: Elongational and Shear Analysis. Proceedings of the SPE Europec featured at 79th EAGE Conference and Exhibition.

[67] Tahir M., Hincapie R.E., Gaol C., Saefken S., Ganzer L. Describing The Flow Behavior Of Smart Water In Micromodels With Wettability Modified Pore Structures. Proceedings of the SPE Latin American and Caribbean Petroleum Engineering, Virtual Conference.

[68] Tahir M., Hincapie R.E., Ganzer L. (2020). Unlocking the Effects of Fluid Optimization on Remaining Oil Saturation for the Combined Sulfate-Modified Water and Polymer Flooding. Energies.

[69] Rock A. (2016). Pore Scale Visualization of Polymer Viscoelasticity Using Particle Tracing in Glass-Silicon-Glass Micormodels That Are Resembling Porous Media. Bachelor’s Thesis.

[70] Al-Shakry B., Skauge T., Shaker Shiran B., Skauge A. (2019). Polymer Injectivity: Investigation of Mechanical Degradation of Enhanced Oil Recovery Polymers Using In-Situ Rheology. Energies.

[71] Al-Shakry B., Skauge T., Shaker Shiran B., Skauge A. (2018). Impact of Mechanical Degradation on Polymer Injectivity in Porous Media. Polymers.

[72] Skauge A., Zamani N., Gausdal Jacobsen J., Shaker Shiran B., Al-Shakry B., Skauge T. (2018). Polymer Flow in Porous Media: Relevance to Enhanced Oil Recovery. Colloids Interfaces.

[73] Sousa P.C., Vega E.J., Sousa R.G., Montanero J.M., Alves M.A. (2017). Measurement of relaxation times in extensional flow of weakly viscoelastic polymer solutions. Rheol. Acta.

[74] Collett C., Ardron A., Bauer U., Chapman G., Chaudan E., Hallmark B., Pratt L., Torres-Perez M.D., Wilson D.I. (2015). A portable extensional rheometer for measuring the viscoelasticity of pitcher plant and other sticky liquids in the field. Plant Methods.

[75] Sachsenheimer D., Hochstein B., Willenbacher N. (2014). Experimental study on the capillary thinning of entangled polymer solutions. Rheol. Acta.

[76] McKinley G.H., Anna S.L., Tripathi A., Yao M. Extensional rheometry of polymeric fluids and the uniaxial elongation of viscoelastic filaments. Proceedings of the 15th International Polymer Processing Society.

[77] Bhardwaj A., Miller E., Rothstein J.P. (2007). Filament stretching and capillary breakup extensional rheometry measurements of viscoelastic wormlike micelle solutions. J. Rheol..

[78] Azad M.S., Dalsania Y.K., Trivedi J.J. (2018). Capillary breakup extensional rheometry of associative and hydrolyzed polyacrylamide polymers for oil recovery applications. J. Appl. Polym. Sci..

[79] Clasen C. (2010). Capillary breakup extensional rheometry of semi-dilute polymer solutions. Korea-Aust. Rheol. J..

[80] Dinic J., Zhang Y., Jimenez L.N., Sharma V. (2015). Extensional Relaxation Times of Dilute, Aqueous Polymer Solutions. ACS Macro Lett..

[81] Del Giudice F., Haward S.J., Shen A.Q. (2017). Relaxation time of dilute polymer solutions: A microfluidic approach. J. Rheol..

[82] Romeo G., D’Avino G., Greco F., Netti P.A., Maffettone P.L. (2013). Viscoelastic flow-focusing in microchannels: Scaling properties of the particle radial distributions. Lab. Chip.

[83] Tahir M., Hincapie R.E., Foedisch H., Abdullah H., Ganzer L. Impact of Sulphates Presence During Application of Smart Water Flooding Combined with Polymer Flooding. Proceedings of the SPE Europec featured at 80th EAGE Conference and Exhibition.

[84] Tahir M., Hincapie R.E., Langanke N., Ganzer L., Jaeger P. (2020). Coupling Microfluidics Data with Core Flooding Experiments to Understand Sulfonated/Polymer Water Injection. Polymers.

[85] Campo-Deaño L., Galindo-Rosales F.J., Pinho F.T., Alves M.A., Oliveira M.S.N. (2011). Flow of low viscosity Boger fluids through a microfluidic hyperbolic contraction. J. Non-Newton. Fluid Mech..

[86] Herbas J.G., Wegner J., Hincapie R.E., Födisch H., Ganzer L., Castillo J.A.D., Mugizi H.M. Comprehensive Micromodel Study to Evaluate Polymer EOR in Unconsolidated Sand Reservoirs. Proceedings of the SPE Middle East Oil & Gas Show and Conference.

[87] Sousa P.C., Pinho F.T., Oliveira M.S.N., Alves M.A. (2010). Efficient microfluidic rectifiers for viscoelastic fluid flow. J. Non-Newton. Fluid Mech..

[88] Schumi B., Clemens T., Wegner J., Ganzer L., Kaiser A., Hincapie R.E., Leitenmueller V. (2019). Alkali/Cosolvent/Polymer Flooding of High-TAN Oil: Using Phase Experiments, Micromodels, and Corefloods for Injection-Agent Selection. SPE Reserv. Eval. Eng..

[89] Gaol C., Wegner J., Ganzer L., Dopffel N., Koegler F., Borovina A., Alkan H. Investigation of Pore-Scale Mechanisms of Microbial Enhanced Oil Recovery MEOR Using Microfluidics Application. Proceedings of the SPE Europec featured at 81st EAGE Conference and Exhibition.

[90] Gaol C.L., Wegner J., Ganzer L. (2020). Real structure micromodels based on reservoir rocks for enhanced oil recovery (EOR) applications. Lab. Chip.

[91] Seguin D., Montillet A., Comiti J., Huet F. (1998). Experimental characterization of flow regimes in various porous media—II: Transition to turbulent regime. Chem. Eng. Sci..

[92] Ergun S., Orning A.A. (1949). Fluid Flow through Randomly Packed Columns and Fluidized Beds. Ind. Eng. Chem..

[93] Tahir M., Hincapie R.E., Langanke N., Ganzer L. Coupling Microfluidics Data with Core Flooding Experiments to Understand Sulphonated/Polymer Water Injection. Proceedings of the Virtual SPE Europec featured at 82nd EAGE Conference and Exhibition.

[94] Al-Saedi H.N., Al-Jaberi S.K., Al-Bazzaz W., Flori R.E. Experimental Study of Flooding both Low Salinity Water and Foam in Sandstone Reservoirs Bearing Heavy Crude Oil. Proceedings of the SPE Gas & Oil Technology Showcase and Conference.

[95] Foedisch H., Abdullah H., Hincapie R.E., Ganzer L. Optimizing Laboratory cEOR Flooding Evaluations to Assess Initial Oil Saturation and Mobility Ratio. Proceedings of the SPE Europec featured at 80th EAGE Conference and Exhibition.

[96] Wu Y., Dong M., Shirif E. (2011). Study of Alkaline/Polymer Flooding for Heavy-Oil Recovery Using Channeled Sandpacks. SPE Reserv. Eval. Eng..

[97] Ringen I., Stiegler H., Nødland O., Hiorth A., Stavland A. Polymer flooding in sandpacks with a dualporosity. Proceedings of the The International Symposium of the Society of Core Analysts.

[98] Aitkulov A., Mohanty K.K. (2019). Investigation of alkaline-surfactant-polymer flooding in a quarter five-spot sandpack for viscous oil recovery. J. Pet. Sci. Eng..

[99] Mall-Gleissle S.E., Gleissle W., Buggisch H., McKinley G.H. (2002). The normal stress behaviour of suspensions with viscoelastic matrix fluids. Rheol. Acta.

[100] Jensen E.A., Christiansen J. (2008). deC. Measurements of first and second normal stress differences in a polymer melt. J. Non-Newton. Fluid Mech..

[101] Nam J.G., Ahn K.H., Lee S.J., Hyun K. (2010). First normal stress difference of entangled polymer solutions in large amplitude oscillatory shear flow. J. Rheol..

[102] Duffy J., Rega C., Kroger M., Jack A., Amin S. (2016). An algebraic approach for determining viscoelastic moduli from creep compliance through application of the Generalised Stokes-Einstein relation and Burgers model. Appl. Rheol..

[103] Pipe C.J., Kim N.J., McKinley G.H. Microfluidic rheometry on a chip. Proceedings of the 4th Annual European Rheology Conference (AERC 2007).

[104] Galindo-Rosales F.J., Campo-Deaño L., Sousa P.C., Ribeiro V.M., Oliveira M.S.N., Alves M.A., Pinho F.T. (2014). Viscoelastic instabilities in micro-scale flows. Exp. Therm. Fluid Sci..

[105] Calvert J.G. (1990). Glossary of atmospheric chemistry terms (Recommendations 1990). Pure Appl. Chem..

[106] (2018). micromod Partikeltechnologie GmbH Downloads. https://www.micromod.de/de/downloads-45.html.

[107] Vik B., Kedir A., Kippe V., Sandengen K., Skauge T., Solbakken J., Zhu D. Viscous Oil Recovery by Polymer Injection; Impact of In-Situ Polymer Rheology on Water Front Stabilization. Proceedings of the SPE Europec featured at 80th EAGE Conference and Exhibition.

[108] Khamees T.K., Flori R.E. Modeling the Combined Effects of Water Salinity and Polymer Rheology on the Performance of Polymer Flooding and In-Depth Gel Treatment. Proceedings of the SPE Western Regional Meeting.

[109] Turkoz E., Perazzo A., Arnold C.B., Stone H.A. (2018). Salt type and concentration affect the viscoelasticity of polyelectrolyte solutions. Appl. Phys. Lett..

[110] Khorsandi S., Qiao C., Johns R.T. Displacement Efficiency for Low Salinity Polymer Flooding Including Wettability Alteration. Proceedings of the SPE Improved Oil Recovery Conference.

[111] Santo A., Muggeridge A. An Investigation into the Benefits of Combined Polymer-Low Salinity Waterflooding. Proceedings of the SPE Asia Pacific Oil and Gas Conference and Exhibition.

[112] Sasaki K. (2002). Charge screening effect in metallic carbon nanotubes. Phys. Rev. B.

[113] Hofmeister F. (1888). Zur Lehre von der Wirkung der Salze: Zweite Mittheilung. http://publikationen.ub.uni-frankfurt.de/frontdoor/index/index/year/2007/docId/15978.

[114] Heemskerk J., Rosmalen R., Janssen-van R., Holtslag R.J., Teeuw D. Quantification of Viscoelastic Effects of Polyacrylamide Solutions. Proceedings of the SPE Enhanced Oil Recovery Symposium.

[115] Seright R.S., Fan T., Wavrik K.E., Balaban R.D.C. (2010). New Insights into Polymer Rheology in Porous Media. SPE J..

[116] Klein J., Kulicke W.M. Polymer-Polymer and Polymer-Solid Interaction and Their Relevance for Polymer Application in Enhanced Oil Recovery. Proceedings of the SPE Oilfield and Geothermal Chemistry Symposium.

[117] Müller A.J., Patruyo L.G., Montano W., Roversi-M. D., Moreno R., Rami’rez N.E., Sa’ez A.E. (1997). Mechanical Degradation of Polymers in Flows Through Porous Media: Effect of Flow Path Length and Particle Size. Appl. Mech. Rev..

[118] Bueche F. (1960). Mechanical degradation of high polymers. J. Appl. Polym. Sci..

[119] Larsen H.A., Drickamer H.G. (1957). Mechanical Degradation and Cross Linking of Polymers by Plastic Deformation at High Pressure. J. Phys. Chem..

[120] Frenkel Статьи Левича—кафедра Электрoхимии. http://www.elch.chem.msu.ru/wp3/index.php/ru/articles/levicharticles/.

[121] Maurer J.J., Harvey G.D. (1987). Thermal degradation characteristics of poly(acrylamide-co-acrylic acid) and poly(acrylamide-co-sodium acrylate) copolymers. Thermochim. Acta.

[122] Ray S., Cooney R. (2012). Thermal Degradation of Polymer and Polymer Composites. Handb. Environ. Degrad. Mater. Second Ed..

[123] Thermal Degradation of Polymer—An Overview | ScienceDirect Topics. https://www.sciencedirect.com/topics/engineering/thermal-degradation-of-polymer.

[124] Rodriguez L., Antignard S., Giovannetti B., Dupuis G., Gaillard N., Jouenne S., Bourdarot G., Morel D., Zaitoun A., Grassl B. A New Thermally Stable Synthetic Polymer for Harsh Conditions of Middle East Reservoirs. Proceedings of the SEG/AAPG/EAGE/SPE Research and Development Petroleum Conference and Exhibition.

[125] Saleh L.D., Wei M., Bai B. (2014). Data Analysis and Updated Screening Criteria for Polymer Flooding Based on Oilfield Data. SPE Reserv. Eval. Eng..

[126] Saboorian-Jooybari H., Dejam M., Chen Z. Half-Century of Heavy Oil Polymer Flooding from Laboratory Core Floods to Pilot Tests and Field Applications. Proceedings of the SPE Canada Heavy Oil Technical Conference.

[127] Mitova V., Grancharov G., Molero C., Borreguero A., Troev K., Rodríguez J. (2013). Chemical Degradation of Polymers (Polyurethanes, Polycarbonate and Polyamide) by Esters of H-phosphonic and Phosphoric Acids. J. Macromol. Sci..

[128] Chemical Degradation—An Overview | ScienceDirect Topics. https://www.sciencedirect.com/topics/materials-science/chemical-degradation.

[129] Lake L.W. (1989). Enhanced Oil Recovery.

[130] Jia R., Yang D., Abd H.B. (2018). 51318-10567-Investigation of the Impact of an Enhanced Oil Recovery Polymer on Microbial Growth and MIC. https://store.nace.org/investigation-of-the-impact-of-an-enhanced-oil-recovery-polymer-on-microbial-growth-and-mic-2.

[131] Al-Moqbali W., Joshi S.J., Al-Bahry S.N., Al-Wahaibi Y.M., Elshafie A.E., Al-Bemani A.S., Al-Hashmi A., Soundra Pandian S.B. Biodegradation of Partially Hydrolyzed Polyacrylamide HPAM Using Bacteria Isolated from Omani Oil Fields. Proceedings of the SPE EOR Conference at Oil and Gas West Asia.

[132] Kolnes J., Nilsson S. Effect of the Core Material on Gelation of a HPAM / Chromium System at High Temperature. Proceedings of the SPE/DOE Improved Oil Recovery Symposium.

[133] PetroWIKI Polymer Waterflooding. https://petrowiki.org/Polymer_waterflooding.

[134] Austad T., Rezaeidoust A., Puntervold T. Chemical Mechanism of Low Salinity Water Flooding in Sandstone Reservoirs. Proceedings of the SPE Improved Oil Recovery Symposium.

